# Trap tales: The influence of red alder stand conditions and forest fragmentation on family-level beetle bycatch diversity

**DOI:** 10.1371/journal.pone.0353780

**Published:** 2026-07-15

**Authors:** Ting Ting Michelle Yim, Sichen Zhou, Debra L. Wertman, Allan L. Carroll

**Affiliations:** Forest Insect Disturbance Ecology Lab, Department of Forest and Conservation Sciences, Faculty of Forestry and Environmental Stewardship, University of British Columbia, Vancouver, British Columbia, Canada; USDA Forest Service Southern Research Station, UNITED STATES OF AMERICA

## Abstract

Beetle (Coleoptera) bycatch from insect traps can provide insights into forest health and biodiversity trends. Red alder, *Alnus rubra*, a nitrogen-fixing tree that supports high invertebrate diversity, is facing alder bark beetle-associated declines and increasing fragmentation in coastal British Columbia (BC), Canada. Despite these threats, no research has investigated beetle diversity or its responses to stand conditions and fragmentation in red alder ecosystems. We hypothesized that in red alder ecosystems, (1) stand conditions influence beetle family bycatch diversity through habitat and food availability, and (2) forest fragmentation influences beetle family diversity by altering habitat connectivity and patch size. We also evaluated how these factors affect abundances of select scolytine predatory families (Salpingidae, Monotomidae, Nitidulidae, Staphylinidae, and Histeridae). Ethanol-baited multiple-funnel traps (*N =* 25) targeting ambrosia beetles were deployed in five red alder-dominated forests in southwestern BC in 2024. Beetle bycatch (*N =* 8226 individuals) was identified to family and determined to be predominantly saproxylic. Family-level Shannon Diversity Index (*H’*), richness, and select abundances were compared between sites, and their relationships with stand conditions and fragmentation analyzed using bivariate models. Site-level differences were observed for *H’*, richness, and abundances, all of which were associated with stand conditions and fragmentation to varying extents. Notably, higher tree density and abundance of dead red alders and lower fragmentation were associated with higher beetle bycatch diversity. Structural heterogeneity, including deadwood availability, and forest continuity likely provided microhabitats supporting saproxylic beetle families in these forests. Examining beetle diversity at the family level allowed us to explore functional associations of saproxylic beetles to environmental conditions across landscapes, although these associations should be interpreted judiciously due to coarse taxonomic resolution and sampling and analytical limitations. Beetle bycatch resolved to family level represents a valuable resource for biodiversity monitoring and conservation management in red alder forests.

## Introduction

Beetles (Coleoptera) represent one of the most taxonomically and functionally diverse insect orders, comprising roughly a quarter of all described animal, fungal and plant species [1,2]. In western North America alone, there are over 130 beetle families encompassing a wide range of life histories, such as phytophagy, mycophagy, xylophagy, and necrophagy [1]. The beetles in these guilds occupy multiple trophic levels and contribute to terrestrial and aquatic ecosystem function. Due to their broad ecological significance and sensitivity to environmental change, beetles are widely regarded as conservation targets and bioindicators of forest health [3,4]. Although several beetle families, such as Carabidae [5] and Silphidae [6], are commonly used in ecological monitoring to assess the impacts of forest disturbances and successional dynamics, the potential for lower taxonomic resolutions of beetle diversity to contribute to studies of forest ecology has been underappreciated [7]. Species-level identifications are often impeded by unknown ecologies and unresolved phylogenies [7,8], as well as resource constraints including time, cost, and taxonomic expertise [8,9]. These challenges are particularly pronounced in speciose beetle families, such as Staphylinidae [10] and Curculionidae [11], where high morphological similarity and incomplete or regionally variable identification keys complicate identifications at fine taxonomic resolutions. Consequently, investigations of diversity at the family level could serve as a practical and informative surrogate to capture community-level patterns of taxonomic and functional diversity of beetles in forest ecosystems (e.g., [12,13]), an approach used for other invertebrates across aquatic and terrestrial ecosystems (e.g., [14–16]) to inform conservation efforts [17].

At the landscape level, forest structural and compositional heterogeneity creates diverse habitats for beetle communities [18]. Forest fragmentation, however, often driven by human developments such as transportation infrastructure and trails, disrupts ecosystems by dividing contiguous forests into smaller, discontinuous patches [19]. Forest fragmentation often leads to insect biodiversity loss and shifts in beetle species composition by reducing habitat patch size and altering microclimatic conditions, ultimately degrading trophic interactions [20,21]. These outcomes are particularly detrimental to forest specialist beetles and species with limited dispersal ability and often result in local extinctions [22]. For the Carabidae community in the temperate urban forests of Japan, for example, fragmented patches support fewer large-bodied species and smaller forest areas are associated with declines in species [23]. Similarly, small forest fragments are characterized by lower species richness and predominantly smaller-bodied individuals of the Scarabaeidae community in the Atlantic forests of Brazil [24]. Many temperate forests in British Columbia (BC), Canada are increasingly fragmented due to expanding human settlement and road development [25]. Understanding how forest fragmentation impacts beetle assemblages is essential to inform conservation strategies in these biodiverse ecosystems.

At the stand level, forest structure shapes beetle community composition [26–28]. Stand structural heterogeneity, generated by vegetation diversity, trees of different age classes, and deadwood availability, influences the functional diversity of beetles. Lassau et al. [26] and Müller et al. [27] found that saprophagous, fungivorous, and predatory beetle guilds each responded differently to stand structural components and suggest that variation in stand conditions promotes greater functional diversity. Large tree diameters and stratified forest structure are able to support higher beetle biomass and diversity, respectively [20]. A higher proportion of deciduous vegetation may also support higher abundances of fungivorous and herbivorous beetles [28].

Red alder, *Alnus rubra* Bong., is the most common broadleaf tree species in the temperate forests of BC [29]. As a pioneer species, it is involved in ecological succession and niche construction in broadleaf–conifer mixed forests [30,31]. Through its symbiosis with *Frankia* bacteria, red alder is able to fix atmospheric nitrogen and produce nitrogen-rich leaf litter, allowing it to colonize nutrient-poor soils and enrich soil fertility on geologically young sites [30,32,33]. The presence of nitrogen-fixing red alder enhances growth of non-nitrogen fixers, such as Douglas-fir, in mixed forest stands and hence indirectly increases the litter nitrogen content of non-fixers, which further improves forest productivity [32]. In mixed red alder–conifer stands, red alder contributes significantly to structural heterogeneity [30], providing diverse microhabitats that support invertebrate richness and biomass, including that of beetles [31]. Despite the ecological significance of red alder, research on beetle diversity in red alder ecosystems is limited and its relationship to stand attributes are largely unexplored.

An often-overlooked source of forest beetle diversity data is trap bycatch, i.e., non-target beetles incidentally captured in traps used for pest monitoring or research programs. In Europe and North America, semiochemical-baited traps are widely used in surveillance programs for bark beetles (e.g., [34]), ambrosia beetles (e.g., [35]), and wood borers (e.g., [36]). These traps frequently capture bycatch due to attraction to kairomones (e.g., by predators) [34], other semiochemicals [37], and visual cues [38]. Ethanol is one of the most commonly used kairomone baits in bark and ambrosia beetle monitoring (e.g., [39,40]); however, it is highly prone to bycatch (e.g., [41]). Likewise, other ecological monitoring studies comparing fermenting baits, such as beer, wine, and sugar (e.g., [42,43]), demonstrate their non-selective attraction of beetles, suggesting potential for bycatch. While trap bycatch is often discarded or stored without being analyzed, these collections represent valuable, ecologically informative subsets of the forest beetle community and are deserving of both scientific and ethical consideration [44]. Studying trap bycatch also informs practical improvements in trapping methods, for example, designing or utilizing more selective lures to minimize unintended captures and promote conservation of ecologically significant species [34,36]. Given the association between red alder and rich invertebrate communities [31], beetle bycatch diversity in insect traps from red alder-dominated forests is likely high and worthy of investigation.

In this study, we examined the family-level diversity of beetle bycatch––incidentally captured in ethanol-baited traps targeting ambrosia beetles––in red alder-dominated forests of the Lower Mainland, BC, Canada. Red alder stands in southwestern BC have evidently experienced decline in recent years, likely attributable to scolytine beetles (i.e., the alder bark beetle, *Alniphagus aspericollis* (LeConte), and ambrosia beetles (Coleoptera: Curculionidae: Scolytinae)) that can attack both stressed and apparently healthy hosts [45–47]. Understanding the potential impacts of red alder decline on beetle community dynamics, specifically functional responses that are best captured at the family-level, is crucial for informing forest management practices that support the conservation of beetle diversity. We evaluated the hypotheses that (1) forest stand conditions in red alder ecosystems influence beetle family bycatch diversity by affecting habitat and food availability, and (2) forest fragmentation influences beetle family bycatch diversity in red alder ecosystems by altering habitat connectivity and patch size. We predicted that (i) favourable stand conditions, such as high dead tree (specifically dead red alder) density, large mean stem diameter, and greater vegetation diversity, would promote higher beetle family bycatch diversity by providing abundant niches and food sources, and (ii) increasing forest fragmentation would decrease beetle family bycatch diversity due to reduced habitat connectivity and patch size. We were also interested in how the abundances of dominant beetle families respond to stand conditions and forest fragmentation; specifically, we focused on the five most abundant families identified in this study that are saproxylic and contain species that prey directly upon scolytines, including Salpingidae, Monotomidae, Nitidulidae, Staphylinidae, and Histeridae [48]. Understanding how families that include these predators respond to forest structure, particularly red alder attributes, and to scolytine abundance may inform management strategies for regulating the red alder scolytine populations that are putatively associated with decline of these ecosystems.

## Materials and methods

### Study sites and beetle collection

**Fig 1:**
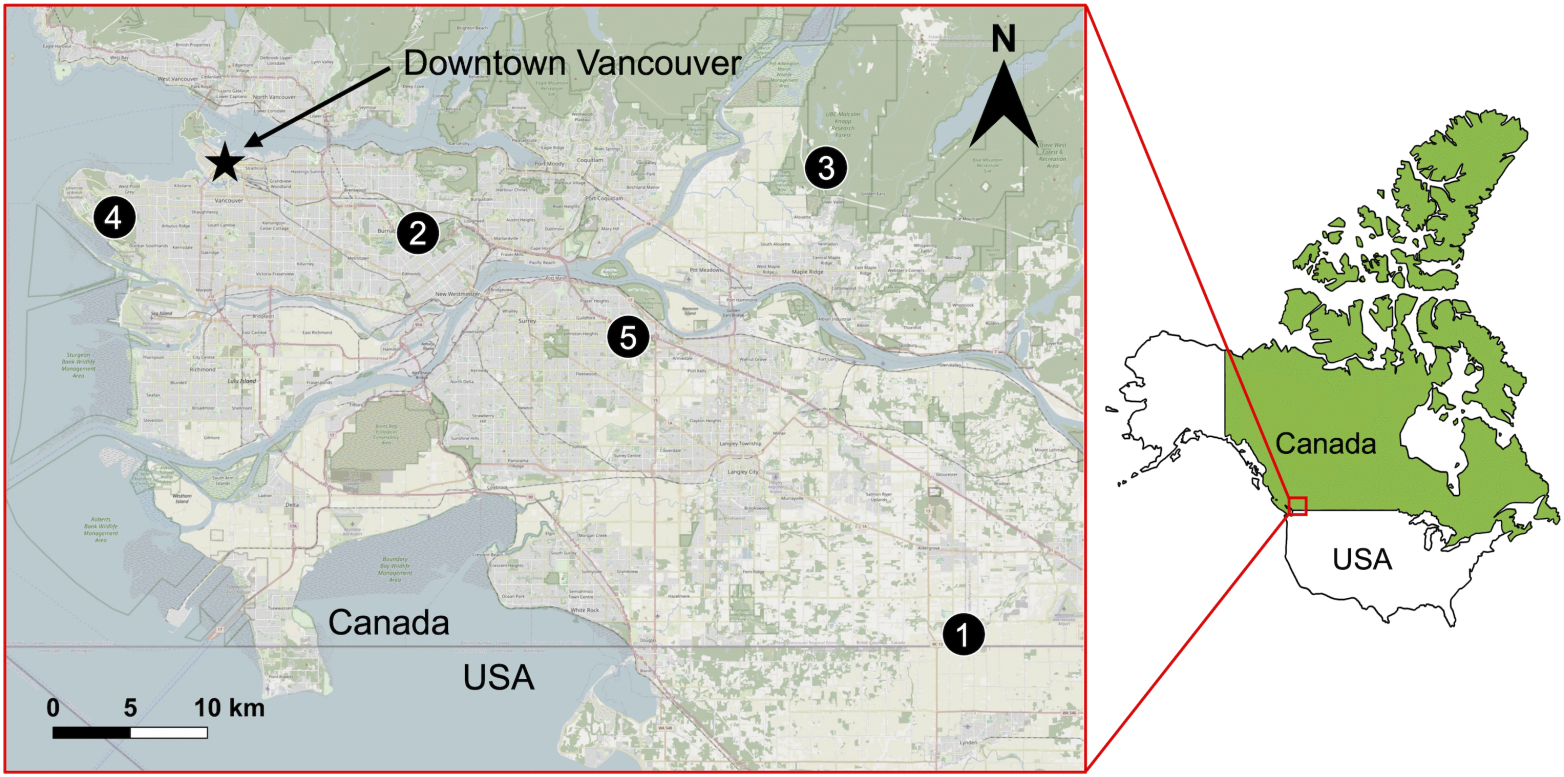
Locations of the five study sites in the Lower Mainland, British Columbia, Canada, where family-level beetle bycatch diversity in red alder, *Alnus rubra*, stands was evaluated. 1 = Aldergrove Regional Park (ARP); 2 = Burnaby Lake Regional Park (BL); 3 = Malcolm Knapp Research Forest (MK); 4 = Pacific Spirit Regional Park (PSP); 5 = Tynehead Regional Park (TRP). North America locator map digitally traced from USGS National Map Viewer (2025, https://apps.nationalmap.gov/viewer/). Inset map adapted from the base map and data from OpenStreetMap and OpenStreetMap Foundation under the Open Data Commons Open Database License, edited and annotated in QGIS 3.40 (2024, qgis.org).

Beetles were collected weekly from 11 February to 3 November 2024 from five red alder-dominated forest sites in the Lower Mainland of BC, Canada: Aldergrove Regional Park (ARP), Burnaby Lake Regional Park (BL), Malcolm Knapp Research Forest (MK), Pacific Spirit Regional Park (PSP), and Tynehead Regional Park (TRP) (Fig 1). Five Lindgren multiple-funnel traps were installed at each site to capture flying beetles [49]. Each trap was equipped with one Ultra-High Release ethanol lure (Synergy Semiochemical Corporation, Delta, BC, Canada), with a release rate of 0.3 mg day^-1^ [50], attached to the fourth funnel from the bottom. Lures were replaced every 4 months. Ethanol is a common attractant for wood borers, bark beetles, and other beetles that rely on stress-induced plant volatiles to find food sources [50,51]. Collection cups attached to the base of the funnel traps were half-filled with propylene glycol (WinterProof Plumbing Antifreeze; Recochem Inc., Montreal, Quebec, Canada) to kill and preserve the beetles upon capture [52]. Within each site, traps were spaced ≥ 20 m apart from one another, hung between two trees that were ≥ 1.5 m apart, with the collection cup positioned > 80 cm aboveground. This study was conducted in compliance with all relevant regulations on publicly owned land in ARP, BL, PSP, and TRP with permits obtained from Metro Vancouver and on privately owned land in MK with a permit obtained from the University of British Columbia.

### Beetle sorting and identification

**Fig 2:**
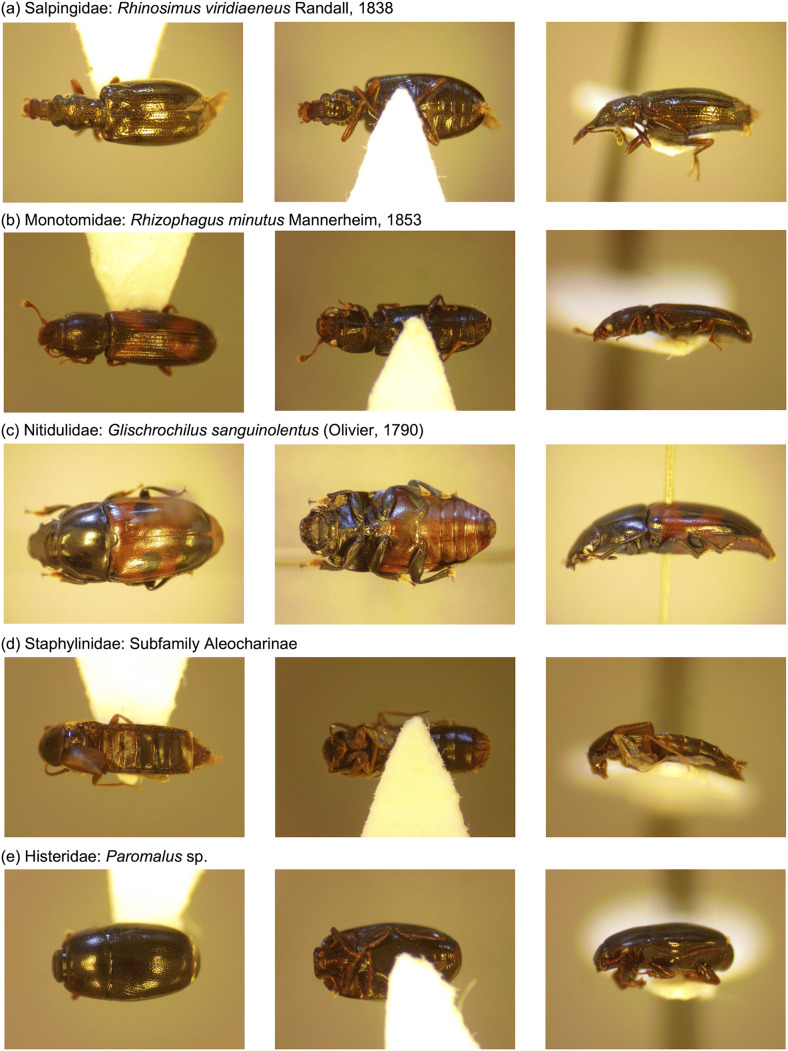
Photographs of representative specimens from five of the most abundant beetle bycatch families identified in this study that are saproxylic and contain scolytine predators captured across five study sites in the Lower Mainland, British Columbia, Canada, where family-level beetle bycatch diversity in red alder, *Alnus rubra*, stands was evaluated. (a) Salpingidae, (b) Monotomidae, (c) Nitidulidae, (d) Staphylinidae, and (e) Histeridae. Specimens are shown in dorsal (left), ventral (centre), and lateral (right) views. Photographs by T. T. M. Yim.

Beetles were stored in propylene glycol in Whirl-Pak Standard Sterilized Bags (Filtration Group, Texas, U.S.) at 4°C following collection. All beetles caught as bycatch were identified to the family level based upon external morphological characteristics, under a dissecting microscope and with the aid of identification guides [1,53–57] and specimen images from the Spencer Entomological Collection Online Database [58]. Key diagnostic features used for family-level identification included but were not limited to the shape, length, and number of antennomeres; position of mouthparts; shape and margin of the pronotum; puncture arrangement of the elytra; size and shape of the elytra and scutellum; abundance and colouration of setae; tarsal formula; presence of tarsal pads and hooks; and overall body form and colouration. Following identification, beetles were preserved in 70% ethanol and stored in 20 mL scintillation vials labelled with the collection site, trap number, collection date, and family name. Representative specimens of each species will be vouchered to the Spencer Entomological Collection (Beaty Biodiversity Museum, Vancouver, BC, Canada) at the stage of forthcoming species-level research. Photographs of selected specimens with key diagnostic traits for each family are provided in Figs 2 and S1.

### Collection of stand condition and forest fragmentation data

An 11.28 m-radius fixed-area plot (area = 400 m^2^) was established at each site to characterize forest stand conditions [59]. All stands were assessed under leaf-on conditions in early- to mid-August 2024. At ARP, MK, PSP, and TRP, the plot centre was positioned at the centroid of a triangle formed among the three most widely positioned funnel traps. At BL, the fifth funnel trap served as the plot centre due to dense Himalayan blackberry growth at the centroid. We defined a tree as a woody perennial with one or more stems and a diameter at breast height (DBH) ≥ 8.0 cm [47], measured at 1.3 m upslope, including live and standing dead individuals. For multi-stemmed trees, if the stems split below the DBH, each stem was recorded as a separate tree; if the stems split above the DBH, they were collectively treated as a single tree. The number, DBH, species, and live/dead status of all standing trees were recorded for each plot. Trees were considered alive if green foliage was present, and dead if foliage was absent for deciduous trees or absent/reddish–brown for coniferous trees. From these data, density, DBH, and diversity metrics were derived for ‘overall stand conditions’ and ‘red alder status’ (Table 1). Because all observed standing dead trees were red alders, dead red alder density and mean dead red alder DBH were considered a component of overall stand conditions, as they were representative of stand structure and health in general. Although standing dead red alders were observed at all survey locations, MK and PSP were the only sites featuring standing dead trees within the fixed area plots, and thus the dead red alder metrics derived from our fixed-area plots are conservative estimates of the amount of dead wood available at each site. The widespread red alder decline caused by the ongoing alder bark beetle epidemic across southwestern BC suggests that additional dead trees were present beyond our survey locations [47].

**Table 1:** Overall stand conditions, red alder status, and forest fragmentation metrics—measured across the five study sites dominated by red alder, *Alnus rubra*, in the Lower Mainland, British Columbia, Canada—assessed for their influences on family-level richness and Shannon Diversity Index (*H’*) of beetle bycatch, as well as the abundances of the five most abundant beetle bycatch families identified in this study that are saproxylic and contain scolytine predators.

Predictor	Calculation	Unit
**Overall stand condition**		
Total scolytine abundance	Sum of scolytine individuals across all funnel traps per site	.
Tree density	No. of living and standing dead tree stems per 400 m^2^ × 25	No. of stems per hectare
Living tree density	No. of living tree stems per 400 m^2^ × 25	No. of stems per hectare
Mean tree DBH^1^	Sum of DBHs / no. of stems for living and standing dead tree stems	cm
Mean living tree DBH^1^	Sum of DBHs / no. of stems for living tree stems	cm
Tree species richness	Species count, inclusive of living and standing dead trees	.
Tree species diversity	Shannon Diversity Index (*H’*) calculated based on equation (1), with *F* replaced by the total number of tree species (*S*) and using raw stem counts as the measure of abundance per species, inclusive of living and standing dead trees	.
**Red alder status**		
Red alder density	No. of living and standing dead red alder stems per 400 m^2^ × 25	No. of stems per hectare
Dead red alder density	No. of standing dead red alder stems per 400 m^2^ × 25	No. of stems per hectare
Living red alder density	No. of living red alder stems per 400 m^2^ × 25	No. of stems per hectare
Mean red alder DBH^1^	Sum of DBHs / no. of stems for living and standing dead red alder stems	cm
Mean dead red alder DBH^1^	Sum of DBHs / no. of stems for standing dead red alder stems	cm
Mean living red alder DBH^1^	Sum of DBHs / no. of stems for living red alder stems	cm
**Forest fragmentation metric**		
Forest area	(Total forest area / 3.14 km^2^) × 100%	%
Number of forest patches	Total no. of forest patches	.
Mean forest patch area	Sum of forest patch areas / no. of forest patches	m^2^
Mean edge-to-area ratio	Sum of (perimeter / area) of forest patches / no. of forest patches	.

Overall stand conditions and red alder status were measured within an 11.28 m fixed-radius plot (area = 400 m^2^). Fragmentation metrics were measured within a 1 km-radius circular plot (area = 3.14 km^2^) centred at the centroid of the triangle formed by the three most widely spaced funnel traps at each site. All observed standing dead trees in the 11.28 m-radius plots were red alders.

^1^DBH refers to diameter at breast height, measured at 1.3 m upslope.

**Fig 3:**
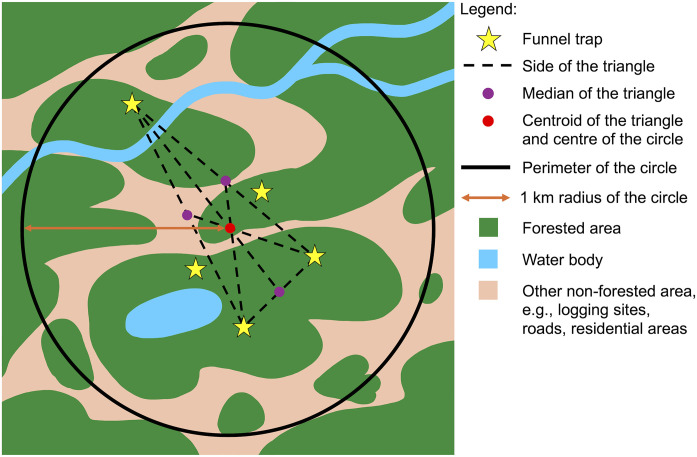
Illustrative example of a 1 km-radius circle used for forest fragmentation measurements. The centre of the circle was defined by the centroid of a triangle formed by three funnel traps farthest apart from one another. Forested areas within the circle were delineated based upon canopy cover to quantify fragmentation (i.e., forest area, number of forest patches, mean forest patch area, and mean edge-to-area ratio) at each of the five study sites in the Lower Mainland, British Columbia, Canada, where family-level beetle bycatch diversity in red alder, *Alnus rubra*, stands was evaluated.

Using Google Earth Pro version 7.3.6.10201 [60], a 1 km-radius circle (area = 3.14 km^2^) was placed over each site to quantify the corresponding extent of forest fragmentation [61] and visually identify primary fragmentation drivers from satellite images (Fig 3). The plot centres used to assess stand conditions (see above) were used as the centres of the circles. Within each circle, forested areas were delineated based upon canopy cover. Water bodies, agricultural areas, recreational areas, ornamental vegetation along roadsides and neighbourhoods, and recently logged and regenerating areas were excluded as traps were placed to capture terrestrial beetles in post-regeneration forests with distinct understory and overstory layers. Forest fragmentation at each site was quantified by percent forest area, number of forest patches, mean forest patch area, and mean edge-to-area ratio [62,63] (Table 1).

### Statistical analyses

#### Beetle family-level diversity metrics.

Two family-level diversity metrics, richness (i.e., total number of families) and Shannon Diversity Index ($H^{\prime }$) [64,65], were used to evaluate relationships between family-level diversity of beetle bycatch and (i) site conditions and (ii) forest fragmentation. Richness and $H^{\prime }$ were calculated from the total captures accumulated throughout the trapping period for each trap. $H^{\prime }$ was calculated as follows [64,66]:


$$\begin{array}{c} H^{\prime }=\;-\sum_{i=\text{1}}^{F}p_{i}lnp_{i} \end{array}$$ (1)

where *F* refers to the total number of beetle families and $p_{i}\;$represents the proportion of individuals in the *i*^*th*^ family. $H^{\prime }$ is defined as the degree of uncertainty in predicting the family of an unknown individual in the ecosystem. As family-level diversity and evenness increase, uncertainty in the prediction also increases, leading to a higher $H^{\prime }$.

#### Comparison of diversity metrics and select family abundances among sites.

Site-level parametric and non-parametric analyses and pairwise comparisons were used to test for differences among the five study sites in family richness, family-level $H^{\prime }$, and abundances of the five most common beetle families identified in this study that contain scolytine predators. Each site was represented by five funnel traps (*n =* 5 per site; total *N*= 25). For $H^{\prime }$, assumptions of normality and homoscedasticity were tested by the Shapiro-Wilk and Levene’s tests, respectively [67,68]. As assumptions were met, one-way ANOVA was used to evaluate differences in $H^{\prime }$ among sites [69], followed by post-hoc Tukey’s Honestly Significant Difference (HSD) tests for pairwise comparisons where *p*(one-way ANOVA) < 0.05 [70]. Since family richness and abundances were ordinal count data and did not follow a normal distribution, the non-parametric Kruskal-Wallis tests were used to assess differences among sites [71], followed by post-hoc Dunn’s tests with Bonferroni correction for pairwise comparisons where *p*(Kruskal-Wallis) < 0.05 [72].

#### Influence of stand conditions and forest fragmentation on family richness, $\begin{array}{c} H^{\prime } \end{array}$ and select family abundances.

We quantified the influence of stand conditions and forest fragmentation metrics using simple linear regressions (SLRs) for family-level $H^{\prime }$ analyses; Conway-Maxwell-Poisson (COM-Poisson) regression models for family richness analyses; and negative binomial generalized linear models (GLMs) for analyses of the abundances of the five select families. Shapiro-Wilk tests and Q-Q plots were used to assess normality for all SLRs [68]. COM-Poisson regressions were used to account for under-dispersion in richness data [73]. Negative binomial GLMs were used to account for over-dispersion in abundance data [74]. Each site was represented by five funnel traps (*n =* 5 per site; total *N* = 25) in the models. Each model included one predictor variable to evaluate its independent effect on the response variable. Mixed-effects models were not applied as predictor variables had only one value per site and thus all predictor variables were confounded by site. Replicate measurements within sites were used to retain within-site variation and identify potential trends and variability. We acknowledge that independence assumptions were not met, increasing the risk of Type I errors. Given the exploratory objective of identifying potential associations relevant to beetle conservation, we prioritized minimizing Type II errors. Analyses were restricted to bivariate models to manage inflated Type I error risk and small sample size, which consequently limited our ability to reliably assess potential collinearity between environmental variables. Accordingly, model comparison and predictor ranking were not performed.

Overall stand conditions (including dead red alder density and mean dead red alder DBH), which were deemed applicable to the wide range of ecologies represented among beetle families, were used for analyses of richness and $H^{\prime }$. To improve model convergence in COM-Poisson regressions evaluating richness, tree density, living tree density predictors, and dead red alder density were scaled by dividing by 10. Overall stand conditions, red alder status, and total scolytine (bark and ambrosia beetle) abundance were used for analyses of the select family abundances to account for the unique ecologies within and among families. Tree density and DBH were used to assess whether the overall beetle bycatch diversity and selected family abundances were influenced by tree size, potentially yielding clues about their food sources or habitat requirements. Tree species richness and diversity were used to evaluate whether beetle family diversity and select family abundances were affected by species heterogeneity. Red alder-specific density and DBH were used to evaluate how the abundances of the predaceous saproxylic families responded to resources associated with red alders.

All forest fragmentation metrics were assessed for their impacts on richness, $H^{\prime }$, and select family abundances, thereby capturing both community-wide and family-specific responses to fragmentation. For richness, mean patch area was scaled by dividing by 10000 in the corresponding COM-Poisson regression to improve model convergence.

#### Statistical computing.

A *p*-value threshold of α = 0.05 and 95% confidence intervals (CI) were used for all data analyses. Data used for statistical analyses were deposited in the Dryad Digital Repository [75]. All analyses and visualizations were conducted using R version 4.4.2 in RStudio version 2024.12.0 + 467 [76,77], aside from visualizations of pairwise comparisons that were produced in Microsoft Excel version 16.95.1 [78]. Shapiro-Wilk, one-way ANOVA, Tukey’s HSD, and Kruskal-Wallis tests, and SLRs were performed using base R functions. Levene’s tests were conducted using the ‘car’ package [79] and Dunn’s tests were performed using the “dunn.test” package [80]. COM-Poisson and negative binomial GLMs were produced using the “glmmTMB” and “MASS” packages, respectively [81,82]. The “dplyr” package [83] was used to calculate the 95% confidence intervals for the COM-Poisson models. Model visualisations were created using the ‘ggplot2’ package [84], with colourblind-friendly palettes from the “viridis” package [85].

## Results

### Summary of beetle bycatch collection

We captured 8226 adult beetles across 50 families as bycatch at the five study sites combined (Figs 2 and S1, S1 Table). The majority of families captured were forest-dwelling and saproxylic, relying on dead or dying wood, wood-associated fungi, or other saproxylic organisms in at least part of their life cycle [86]. Many of the saproxylic families identified were mycophagous, including the three most abundant families captured in our study, i.e., Salpingidae (25.3% of the total catch) [87], Latridiidae (24.1%) [88], and Monotomidae (10.3%) [89]. All remaining families individually comprised less than 10% of the total catch, with Nitidulidae, Staphylinidae, and Histeridae accounting for 5.3%, 4.0%, and 2.5%, respectively. Beyond the five most common families that contain scolytine predators (Salpingidae, Monotomidae, Nitidulidae, Staphylinidae, and Histeridae), additional families known to prey upon scolytines, such as Cleridae and Pythidae, were also present but infrequently captured [48]. Among the sites, MK contributed the largest number of bycatch (27.6%), followed by BL (25.2%), PSP (18.2%), TRP (15.8%), and ARP (13.3%).

### Overview of overall stand conditions, red alder status, and forest fragmentation metrics

Overall stand conditions and red alder status varied substantially among sites (Table 2). Large numbers of scolytine beetles were present at every site; however, ARP produced only ca. one-third of the scolytines compared to each of the other sites. Additionally, tree composition was variable, with BL and TRP comprising entirely deciduous species (predominantly red alder, *Acer* spp., and *Betula* spp.), whereas the remainder of the sites included several conifer species (*Pseudotsuga menziesii*, *Thuja plicata*, and *Tsuga heterophylla*) in addition to the aforementioned deciduous species. Furthermore, the number of tree species within each site-level plot ranged from 1 to 7, with the TRP plot comprised of only red alders. Thus, the overall richness and diversity of trees were also variable among sites. In terms of stand structure, the density of trees was highly variable, with PSP having three times the density at TRP. Not surprisingly, sites with lower tree density generally had larger trees; for example, red alders at ARP, BL, and TRP were approximately twice the size of those at MK and PSP. Overall tree size and living tree size were nearly identical within sites (Table 2). Given that the two metrics were effectively redundant, we focused on overall tree DBH hereafter.

**Table 2:** Summary of stand condition, red alder status, and forest fragmentation data per site—measured across the five study sites dominated by red alder, *Alnus rubra*, in the Lower Mainland, British Columbia, Canada—assessed for their influences on family-level richness and Shannon Diversity Index (*H’*) of beetle bycatch, as well as the abundances of the five most abundant beetle bycatch families identified in this study that are saproxylic and contain scolytine predators.

Predictor	ARP	BL	MK	PSP	TRP
**Overall stand condition**					
Total scolytine abundance	10350	30678	34871	30759	34987
Tree density (stems ha^-1^)^1,3^	475	675	925	1075	350
Living tree density (stems ha^-1^)	475	675	475	775	350
Mean tree DBH (cm)^1,2,3^	28.4 ± 3.0	20.9 ± 2.7	16.4 ± 0.7	21.9 ± 2.5	36.9 ± 3.4
Mean living tree DBH (cm)^2^	28.4 ± 3.0	20.9 ± 2.7	15.6 ± 1.1	22.6 ± 3.5	36.9 ± 3.4
Tree species richness^1,3^	7	6	4	7	1
Tree species diversity^1,3^	1.58	1.49	0.88	1.50	0
**Red alder status**					
Red alder density (stems ha^-1^)^1,3^	225	125	650	450	350
Dead red alder density (stems ha^-1^)^3^	0	0	450	300	0
Living red alder density (stems ha^-1^)	225	125	200	150	350
Mean red alder DBH (cm)^1,2,3^	36.3 ± 1.9	36.0 ± 3.8	18.0 ± 0.6	19.7 ± 1.1	36.9 ± 3.4
Mean dead red alder DBH (cm)^2,3^	-	-	17.3 ± 0.7	20.2 ± 1.4	-
Mean living red alder DBH (cm)^2^	36.3 ± 1.9	36.0 ± 3.8	19.4 ± 1.0	18.7 ± 2.1	36.9 ± 3.4
**Forest fragmentation metric**					
Forest area (%)	49.20	29.87	85.73	42.97	62.49
Number of forest patches	19	10	12	2	24
Mean forest patch area (m^2^)	81304 ± 59292	93800 ± 42316	224324 ± 72069	674580 ± 265278	81753 ± 47373
Mean edge-to-area ratio	0.0819 ± 0.0118	0.0556 ± 0.0152	0.0481 ± 0.0155	0.00743 ± 0.00186	0.0772 ± 0.0107

Stand conditions were measured within 11.28 m-radius fixed-area plots (area = 400 m^2^), while fragmentation metrics were measured within 1 km-radius circles (area = 3.14 km^2^) placed over each site centred at the centroid of the triangle formed by the three most widely spaced funnel traps at each site. Mean values are presented with standard errors (± SE). Site abbreviations: ARP = Aldergrove Regional Park, BL = Burnaby Lake Regional Park, MK = Malcolm Knapp Research Forest, PSP = Pacific Spirit Regional Park, TRP = Tynehead Regional Park. See Table 1 for predictor calculation details.

^1^Inclusive of living and standing dead trees.

^2^DBH refers to diameter at breast height, measured at 1.3 m upslope.

^3^All observed standing dead trees in the 11.28 m-radius plots were red alders.

The extent of forest fragmentation and types of fragmentation drivers also varied among sites (Table 2). At ARP, fragmentation was predominantly associated with agricultural and recreational land uses, whereas BL, PSP, and TRP were fragmented by residential and commercial land uses. PSP had the most continuous forest and was the least fragmented despite having relatively small total forest area and proximity to human infrastructure and settlements. BL had the smallest total forest area, as much of the site is occupied by a large lake and surrounding marshes dominated by herbaceous vegetation and scattered shrubs. BL still included more intact forest than ARP and TRP, which had the most pronounced fragmentation evidenced by numerous discontinuous and comparatively small forest fragments. Although MK was the site most remote from urban areas, it showed moderate fragmentation from meandering forestry roads and young even-aged stands indicative of forestry activities that produced discontinuous forest patches. No evidence of forestry activity was observed at any sites aside from MK.

### Comparison of diversity metrics and select family abundances among sites

**Fig 4:**
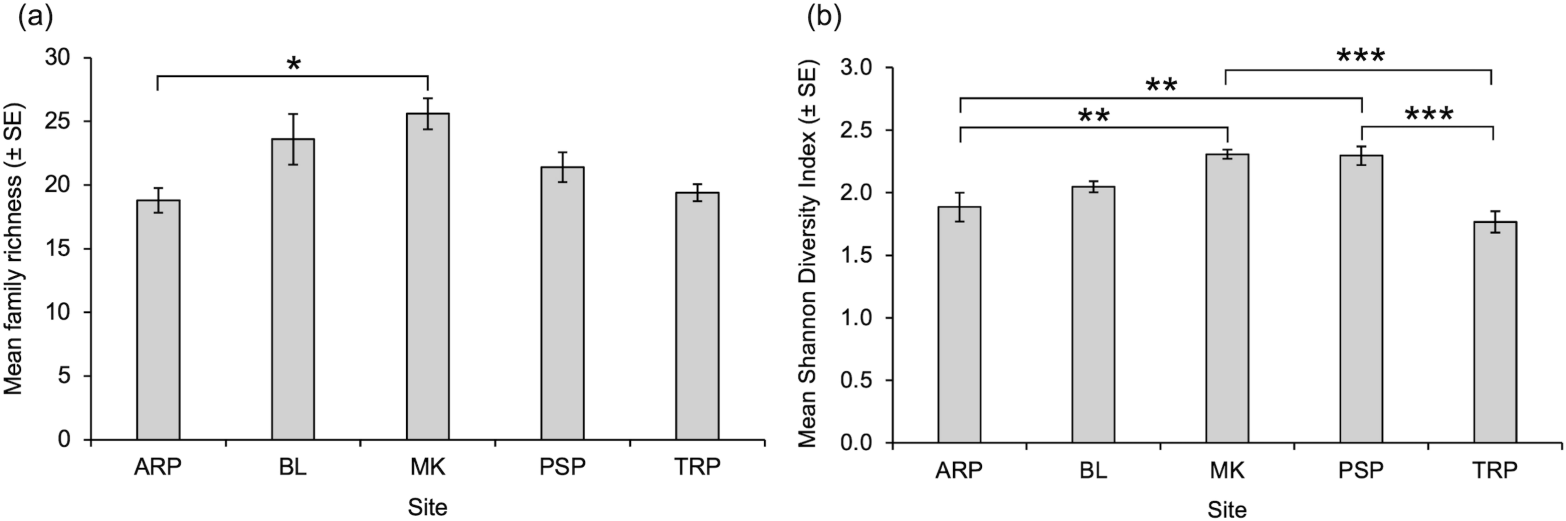
Pairwise site comparison results of family-level (a) richness and (b) Shannon Diversity Index ($\begin{array}{c} H^{\prime } \end{array}$) of beetle bycatch among the five study sites (*n* = 5 traps per site) dominated by red alder, *Alnus rubra*, in the Lower Mainland, British Columbia, Canada. Richness was compared using Dunn’s tests with Bonferroni correction following a significant Kruskal-Wallis test (*p* < 0.05). $H^{\prime }$ was compared using Tukey’s Honestly Significant Difference (HSD) tests following a significant one-way ANOVA test (*p* < 0.05). Levels of significances are denoted by asterisks: *p* < 0.05 *, p < 0.01 **, and *p* < 0.001 ***. Site abbreviations: ARP = Aldergrove Regional Park, BL = Burnaby Lake Regional Park, MK = Malcolm Knapp Research Forest, PSP = Pacific Spirit Regional Park, TRP = Tynehead Regional Park.

As expected, both family richness and diversity ($H^{\prime }$) of beetles differed among sites, and the two metrics were not redundant (Fig 4). MK had the highest richness, notably exceeding ARP (Fig 4a). Despite having the smallest forest area, BL also showed relatively high richness compared to other sites (Fig 4a). Although PSP had fewer families than BL (Fig 4a), its beetle assemblage was more diverse, comparable to that of MK (Fig 4b). MK and PSP— the only sites with dead red alders and the highest tree density within plots—had substantially higher diversity than the other sites that lacked dead trees and had lower tree densities, especially ARP and TRP (Fig 4b).

**Fig 5:**
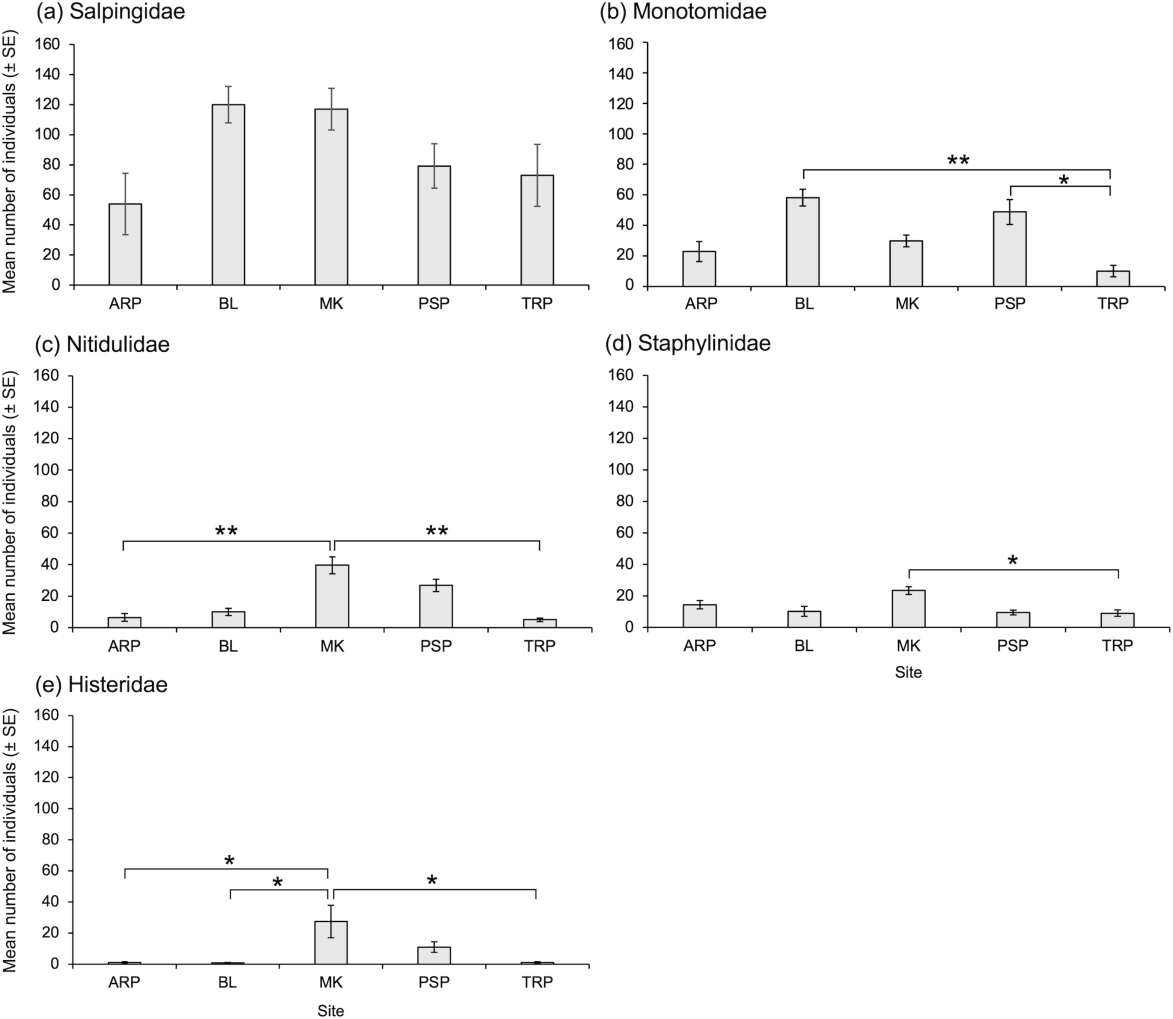
Pairwise comparison results for the five most abundant beetle bycatch families identified in this study that are saproxylic and contain scolytine predators among the five study sites (*n* = 5 traps per site) dominated by red alder, *Alnus rubra*, in the Lower Mainland, British Columbia, Canada. (a) Salpingidae, (b) Monotomidae, (c) Nitidulidae, (d) Staphylinidae, and (e) Histeridae. Dunn’s tests with Bonferroni correction were performed following significant Kruskal Wallis tests (*p* < 0.05). Salpingidae was excluded from the post-hoc analyses as no significant differences were detected among sites. Levels of significances are denoted by asterisks: *p* < 0.05 *, p < 0.01 **, and *p* < 0.001 ***. Site abbreviations: ARP = Aldergrove Regional Park, BL = Burnaby Lake Regional Park, MK = Malcolm Knapp Research Forest, PSP = Pacific Spirit Regional Park, TRP = Tynehead Regional Park.

Among the select families containing scolytine predators, Salpingidae was the most abundant (Fig 5a), followed by Monotomidae and Nitidulidae (Fig 5b, c). Monotomids were the most abundant at BL and PSP (Fig 5b), i.e., the two sites with the smallest total forest area (Table 2). Moreover, Nitidulidae, Staphylinidae, and Histeridae were the most common at MK (Fig 5c–e), i.e., the site with the largest total forest area and highest density of red alders (Table 2).

### Influence of overall stand conditions, red alder status, and forest fragmentation on beetle family diversity metrics and select family abundances

**Fig 6:**
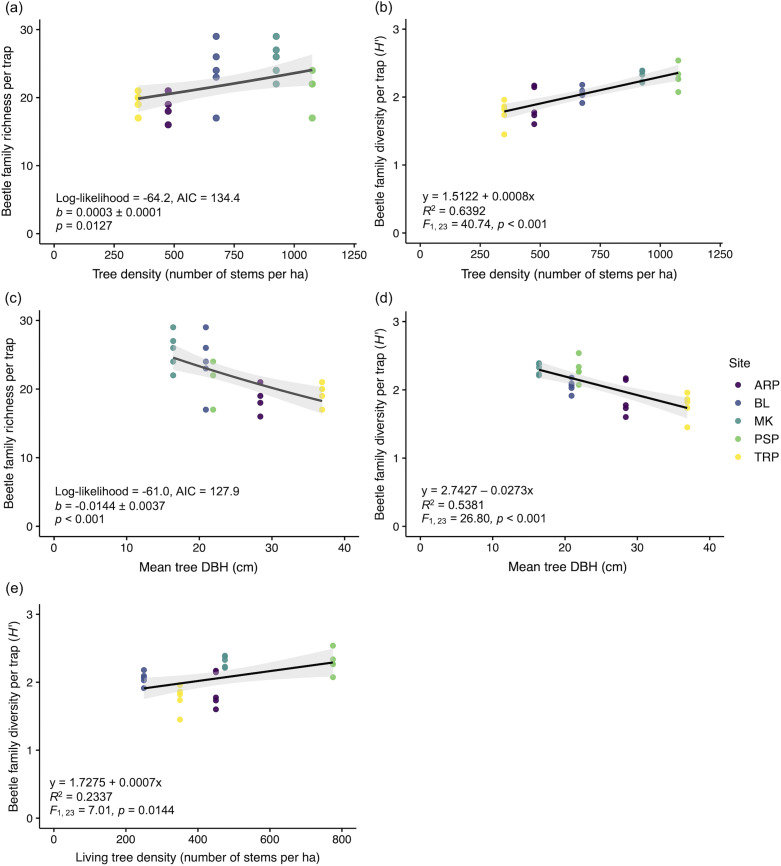
Effects of overall stand conditions on family-level richness and Shannon Diversity Index ($H^{\prime }$) of beetle bycatch across the five study sites (*n* = 5 traps per site) dominated by red alder, *Alnus rubra*, in the Lower Mainland, British Columbia, Canada. Both richness and *H’* were affected by (a, b) tree density (inclusive of live and standing dead trees), and (c, d) mean tree diameter at breast height (DBH, measured at 1.3 m upslope; inclusive of live and standing dead trees). *H’* was affected by (e) living tree density. Log-likelihood, Akaike’s Information Criterion (AIC), standardised regression coefficient (*b*) ± SE, and *p-*value of the Conway-Maxwell-Poisson (COM-Poisson) models for richness are provided. Model equation, *R*^2^, *F*-statistic, and *p*-value of the simple linear regressions (SLRs) for $H^{\prime }$ are provided. Site abbreviations: ARP = Aldergrove Regional Park, BL = Burnaby Lake Regional Park, MK = Malcolm Knapp Research Forest, PSP = Pacific Spirit Regional Park, TRP = Tynehead Regional Park. Regression lines (black) are bounded by 95% CIs (grey).

Beetle family richness and diversity increased with overall tree density (Fig 6a, b) and correspondingly decreased with larger tree size (Fig 6c, d). Diversity also increased with the density of living trees (Fig 6e), although richness was not affected by this metric. As scolytine beetle abundance increased, so did the abundances of Salpingidae, Nitidulidae, and Histeridae (Table 3). Total tree density was associated with increases in monotomids, nitidulids, and histerids, whereas only nitidulids and histerids increased with the density of living trees. Larger tree size was associated with decreased abundances of Monotomidae, Nitidulidae, Staphylinidae, and Histeridae, while only monotomids increased with tree richness and diversity. The abundance of Salpingidae was largely insensitive to stand conditions except scolytine beetle abundance.

**Table 3:** Summary of influences of overall stand condition, red alder status, and forest fragmentation metrics on the abundances of the five most abundant beetle bycatch families identified in this study that are saproxylic and contain scolytine predators across the five study sites dominated by red alder, *Alnus rubra*, in the Lower Mainland, British Columbia, Canada.

Predictor	Salpingidae	Monotomidae	Nitidulidae	Staphylinidae	Histeridae
**Overall stand condition**					
Total scolytine abundance	**+**	.	**+**	.	**+**
Tree density^1,3^	.	**+**	**+**	.	**+**
Living tree density	.	.	**+**	.	**+**
Mean tree DBH^1,2,3^	.	**–**	**–**	**–**	**–**
Tree species richness^1,3^	.	**+**	.	.	.
Tree species diversity^1,3^	.	**+**	.	.	.
**Red alder status**					
Red alder density^1,3^	.	.	**+**	**+**	**+**
Dead red alder density^3^	.	.	**+**	**+**	**+**
Living red alder density	.	**–**	**–**	.	**–**
Mean red alder DBH^1,2,3^	.	.	**–**	**–**	**–**
Mean dead tree DBH^2,3^	.	.	**+**	.	**+**
Mean living red alder DBH^2^	.	.	**–**	.	**–**
**Forest fragmentation metric**					
Forest area	.	**–**	**+**	**+**	**+**
Number of forest patches	.	**–**	**–**	.	**–**
Mean forest patch area	.	.	**+**	.	**+**
Mean edge-to-area ratio	.	**–**	**–**	.	**–**

All predictors were analyzed on their original scale based upon negative binomial generalised linear models (GLMs) with one predictor per model. A “**+**” indicates a positive relationship (*p* < 0.05), “**–**” indicates a negative relationship (*p* < 0.05), and “.” denotes a non-significant relationship (*p* > 0.05). See S2 Table for a summary of statistical outputs.

^1^Inclusive of living and standing dead trees.

^2^DBH refers to diameter at breast height, measured at 1.3 m upslope.

^3^All observed standing dead trees in the 11.28 m-radius plots were red alders.

**Fig 7:**
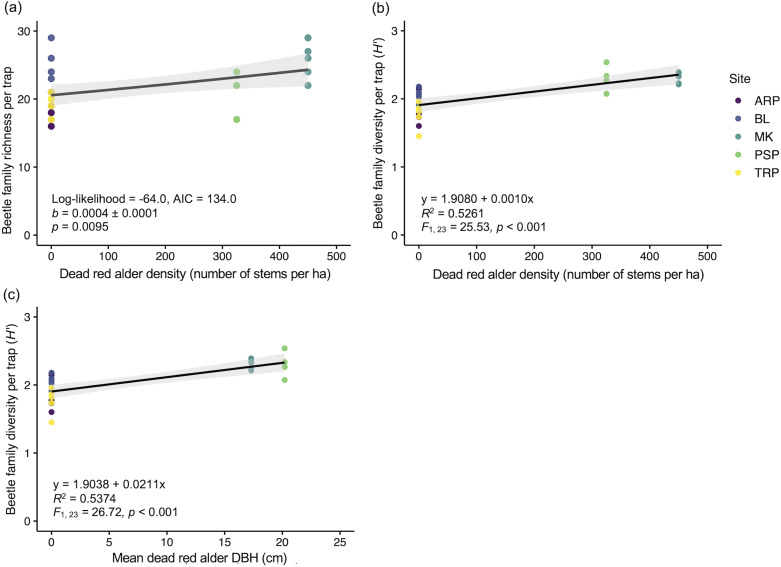
Effects of dead red alders on family-level richness and Shannon Diversity Index ($\begin{array}{c} H^{\prime } \end{array}$) of beetle bycatch across the five study sites (*n* = 5 traps per site) dominated by red alder, *Alnus rubra*, in the Lower Mainland, British Columbia, Canada. Both (a) richness and (b) $H^{\prime }$ were affected by dead red alder density. (c) $H^{\prime }$ was affected by dead red alder DBH (DBH, measured at 1.3 m upslope). Log-likelihood, Akaike’s Information Criterion (AIC), standardised regression coefficient (*b*) ± SE, and *p-*value of the Conway-Maxwell-Poisson (COM-Poisson) model for richness are provided. Model equation, *R*^2^, *F*-statistic, and *p*-value of the simple linear regressions (SLRs) for $H^{\prime }$ are provided. Site abbreviations: ARP = Aldergrove Regional Park, BL = Burnaby Lake Regional Park, MK = Malcolm Knapp Research Forest, PSP = Pacific Spirit Regional Park, TRP = Tynehead Regional Park. Regression lines (black) are bounded by 95% CIs (grey).

Both richness and diversity of beetle families increased with dead red alder density (Fig 7a, b); however, only diversity was positively associated with dead red alder size (Fig 7c). Among the scolytine predatory families, the abundances of Nitidulidae, Staphylinidae, and Histeridae also increased with the density and size of dead red alders (Table 3). Conversely, these abundances generally decreased with denser and larger living red alders (Table 3). Salpingidae abundance was not affected by any of the red alder status metrics.

**Fig 8:**
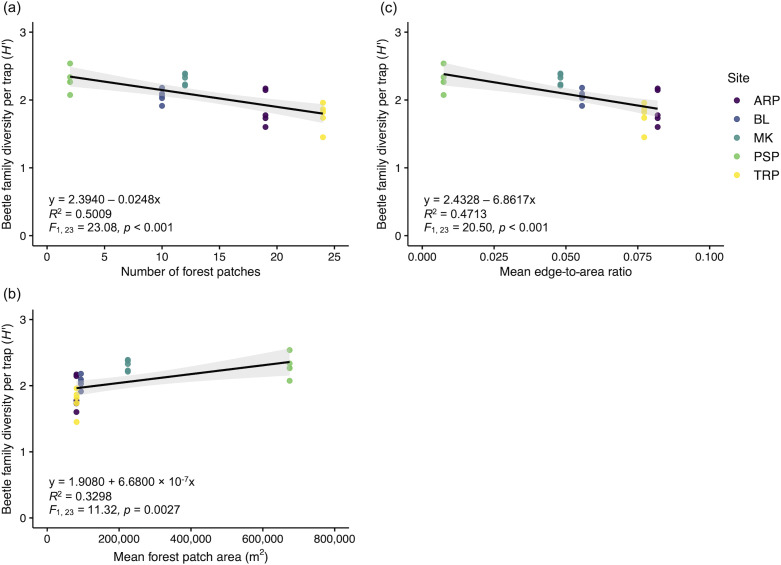
Effects of forest fragmentation metrics on family-level Shannon Diversity Index ($\begin{array}{c} H^{\prime } \end{array}$) of beetle bycatch across the five study sites (*n* = 5 traps per site) dominated by red alder, *Alnus rubra*, in the Lower Mainland, British Columbia, Canada. (a) Number of forest patches, (b) mean edge-to-area ratio, and (c) mean forest patch area. Model equation, *R*^2^, *F*-statistic, and *p*-value of the simple linear regressions (SLRs) are provided. Site abbreviations: ARP = Aldergrove Regional Park, BL = Burnaby Lake Regional Park, MK = Malcolm Knapp Research Forest, PSP = Pacific Spirit Regional Park, TRP = Tynehead Regional Park. Regression lines (black) are bounded by 95% CIs (grey).

Beetle family diversity and scolytine predatory family abundances were negatively affected by forest fragmentation (Fig 8, Table 3). Beetle family diversity and the number of monotomids, nitidulids, and histerids declined with more discontinuous forest fragments and longer forest edges (Fig 8a, b, Table 3). Conversely, these metrics increased with the area of forest fragments and total forest area (Fig 8c, Table 3). Counterintuitively, the abundance of Monotomidae decreased with larger total forest area despite being sensitive to fragmentation (Table 3). Beetle richness and the abundance of Salpingidae were not influenced by fragmentation.

## Discussion

Consistent with our hypotheses, stand conditions indicative of greater habitat availability tended to support higher beetle family diversity and abundances, whereas fragmentation tended to reduce beetle family diversity and abundances in the five red alder ecosystems examined in this study. Family diversity, richness, and beetle abundances were each associated with different subsets of stand conditions and forest fragmentation metrics, apparently reflecting distinct beetle assemblages among red alder-dominated forest. Consistent with the inherent selectivity of our sampling method (i.e., ethanol-baited multiple-funnel traps), the following interpretations are largely restricted to terrestrial saproxylic beetles capable of flight within the forest understory.

Saproxylic beetle family diversity in red alder ecosystems appears to be driven by the amount of dead wood available as both habitat and a food reservoir. Although our fixed-area plots did not consistently capture the standing dead red alders present at all study sites, increases in family richness and diversity with the density and size of standing dead red alders aligns with well-established evidence that larger volumes of dead wood promote higher saproxylic beetle richness and diversity [90–92]. Standing dead trees may also increase structural connectivity among dead stems and branches, facilitating dispersal and resource-finding opportunities for saproxylic beetles to increase richness [93]. Furthermore, larger dead wood is likely to favour fungal growth and thus support rich fungal communities that benefit mycophages and increase saproxylic beetle richness [94,95]. We suggest that fungal diversity could be one of the pathways through which dead-wood volume enhances beetle family diversity. Complex beetle–fungi interactions, where beetles facilitate the introduction of diverse fungal species into wood [96] that subsequently provide food for mycophages and/or enhance nutritional quality of wood for xylophages [97–99], may create a positive feedback loop that reinforces high beetle family diversity. Larger volumes of dead wood may also increase the range of microhabitats with unique microclimatic conditions (e.g., distinct light and humidity conditions) available to sustain diverse saproxylic beetle assemblages [100,101]. Abundances of Monotomidae, Nitidulidae and Staphylinidae generally exhibited the same positive responses to the availability of dead trees, consistent with their saproxylic and partly mycophagous lifestyles [102–104], further suggesting an important association between saproxylic beetle populations and dead wood in red alder forests. While small-scale dead wood inputs such as bark fragments and fallen branches are provided by living trees at high stand densities with intense competition [105], large living trees are often vigorous with reduced risk of injury at low densities [106], hence plausibly producing lower amounts of dead materials and supporting lower beetle family diversity. Because living trees were far more abundant than dead trees at all sites, the positive effect of dead tree size was obscured when overall mean tree size (inclusive of live and dead stems) within stands was considered. The discrepancy between the effects of living and dead trees underscores the importance of distinguishing between living and dead trees when interpreting the influence of varying stand conditions on diversity-related metrics, as grouping living and dead trees together for analysis may obscure their respective magnitudes and/or directions of influence.

The responses by families containing scolytine predatory species to increasing scolytine abundance may be attributable to a direct numerical response to high prey abundance and/or an indirect response associated with scolytine-caused red alder mortality at these sites [47] that could have increased habitat for saproxylic beetles [97,98] and facilitated the proliferation of fungi for mycophagous species [99,107]. The extent to which these non-mutually exclusive mechanisms may contribute to the variation in abundances of our focal families appears to depend on the conditions of each site and the general life histories of each family. The abundances of salpingids, nitidulids, and histerids were directly related to scolytine abundance. Although Salpingidae is saproxylic and includes species with mycophagous stages [87], the only stand condition related to its abundance in this study was the availability of potential scolytine prey, suggesting a direct density-dependent response by predatory species in this family. Nitidulidae is similarly saproxylic and inclusive of mycophagous species [103], but its abundance was related to the density of dead red alders in addition to scolytine abundance, suggesting that nitidulid species may have responded both directly to the availability of prey and indirectly to the availability of the saproxylic habitat. Even though histerid abundance was positively related to dead red alder density and scolytine abundance, this predatory family is non-mycophagous [108], suggesting that the abundance of this family was an outcome of a direct density-dependent response to prey species associated with dead wood. By contrast, the abundance of staphylinids was unrelated to scolytine abundance but positively related to the availability of dead red alder, suggesting that the staphylinid species captured in this study were likely largely non-predatory and responded to increased availability of saproxylic habitat. The abundance of monotomids was unrelated to either scolytine abundance or dead red alder density and may have been dependent upon other aspects of the ecosystem.

Lack of associations between beetle family diversity metrics and tree species richness and diversity in our study is inconsistent with existing evidence of positive relationships between beetle diversity and tree species diversity (e.g., [109,110]). Since most beetles captured in this study were from saproxylic families that often do not have strict host species requirements for habitat [111], beetle family diversity was likely driven by the density and size of dead trees rather than the taxonomic diversity of vegetation. An exception was Monotomidae, which showed higher abundance with greater tree species richness and diversity, suggesting that although overall beetle family diversity in red alder ecosystems may not depend upon host tree diversity, certain families could still benefit from a higher vegetation diversity.

Negative responses of beetle family diversity and abundances to forest fragmentation are congruent with studies demonstrating the detrimental impacts of habitat fragmentation on various beetle communities (e.g., [22,112,113]). Red alder ecosystems at sites such as ARP and TRP consisted of small and isolated forest patches characterized by long edges and limited interior forest area due to intensive surrounding land uses (i.e., agriculture, residential, and/or recreation), creating conditions that were presumably unfavourable for many forest-dwelling beetle families and resulted in lower saproxylic beetle diversity. On the other hand, despite moderate forestry activity at MK and the residential areas surrounding PSP, both sites retained relatively intact forests with fewer roads and forest edges and, as our results suggest, may thereby sustain higher saproxylic beetle diversity. Similarly, increasing forest fragmentation was associated with reduced abundances, with Nitidulidae and Histeridae showing the strongest declines in relation to fragmentation metrics among the select families; consistent with this trend are the relatively higher abundances for these families at the less-fragmented MK. This result also aligns with Seibold et al. [114] who found that saproxylic beetles dependent upon larger tree diameters or broadleaved hosts are particularly vulnerable to extinction under habitat degradation. Since both beetle family richness and diversity increased with dead red alder size (DBH) in our study, many of the saproxylic families identified likely rely upon broadleaved red alder substrates to some extent and are therefore threatened by fragmentation. Further, with edge effects able to penetrate deep into forest patches, forest fragmentation may also reduce the population sizes of interior forest-adapted beetle species [115]. Interestingly, although the abundance of Monotomidae decreased with higher edge-to-area ratio and number of forest patches, it also declined with increasing total forest area, indicating that some monotomid species may utilize or tolerate non-forested habitats, while also suggesting that forest area may not be a particularly informative forest fragmentation metric for this family.

That family richness was not influenced by forest fragmentation in our study is inconsistent with previous studies that identified declines in species richness with increased forest fragmentation [112,113,116]. A plausible explanation for this discrepancy is that the impacts of forest fragmentation are expressed more strongly at the species level than the family level, as tolerance to fragmentation is highly species-specific [117] and, in some cases, even varies within species [118]. Species turnover processes induced by fragmentation may not be obvious when using family richness as the diversity metric. For example, the loss of fragmentation-sensitive species may be offset by the persistence or expansion of fragmentation-tolerant species of the same family, particularly those adapted to edge habitats, thereby maintaining family-level richness despite underlying shifts in species composition [119,120]. Therefore, assessing the effects of fragmentation at finer taxonomic resolutions, i.e., genus and species, would likely provide insights into within-family compositional changes. More complex indicators of fragmentation—such as matrix quality of forest patches [116], edge effects within ecotones or at abrupt forest-urban transitions [13,115], and dispersal limitations arising from both fragmentation and beetle life history traits [118]—were not quantified in our study, thereby limiting causal interpretation. Nevertheless, our results indicate that relatively simple metrics were sufficient to detect potential effects of fragmentation on saproxylic beetles in southwestern BC red alder ecosystems.

High rates of red alder mortality in southwestern BC in recent years have been attributed to epidemic populations of the alder bark beetle and are suggestive of overall decline in these ecosystems [46,47]. Alder bark beetles can cause mortality to a large proportion of red alder trees in a given stand. Indeed, within some of our study sites, mortality rates ranged from 2–12 stems hectare^-1^ year^-1^ in the several years preceding our investigation [46]. Our results suggest that an abrupt input of dead red alder trees will cause a rapid rise in saproxylic diversity, which was evident at MK and PSP in 2024. If red alder decline continues in these ecosystems, we expect saproxylic beetle family diversity to remain high but eventually fall off without a substantial continuous input of dead wood [121]. Given the rapid decomposition of red alders [122], the saproxylic beetle assemblage will likely also undergo a rapid compositional shift throughout this process [121].

Several methodological limitations should be considered when interpreting and generalizing our findings on beetle bycatch diversity in red alder ecosystems. First, captures from ethanol-baited multiple-funnel traps were biased toward flying terrestrial saproxylic beetles and may have underrepresented other functional groups, such as aquatic and semi-aquatic families, e.g., Scirtidae, Hydrophilidae, Hydraenidae, and Dystiscidae [123], which were likely incidentally captured due to the presence of freshwater bodies at all sites. Ground-dwelling families, such as Carabidae and Silphidae [124,125], were also rarely collected as many of these species are flightless and inhabit the forest floor rather than microhabitats around trap height in the understory. Second, analyses using diversity indices at a coarse taxonomic resolution, i.e., family level, may mask species turn-over and inflate perceived site-level diversities [126]. However, our primary objective was to compare beetle diversity among structurally distinct red alder-dominated forests to identify broad ecological relationships, and thus our findings are intended to reflect broad-scale saproxylic community trends rather than fine-scale, species-specific associations with stand conditions and forest fragmentation. Third, the relatively small number of sites and fixed-area plot configuration may not have fully captured variation in dead red alder availability consistently across sites nor landscape heterogeneity across the geographical range of red alders in southwestern BC. Consequently, these factors restricted our ability to differentiate the effects of correlated environmental variables, including potential collinearity between stand conditions and fragmentation metrics, which led us to utilize bivariate models. In addition, a degree of pseudoreplication may be present in our models as traps within sites share the same environmental predictor values. Due to potential collinearity and pseudoreplication, predictors were not formally ranked or compared across models, and all results should be considered as exploratory. Finally, this study represents a temporal snapshot and does not account for interannual variability in beetle diversity, which is prone to fluctuations induced by climate and stochasticity [127,128].

Overall, our results indicate that saproxylic beetle family diversity is associated with forest stand conditions and susceptible to forest fragmentation in red alder ecosystems of the Lower Mainland, BC. Even within red alder forests of similar overstory composition and comparable ecosystem types, site-specific differences may generate distinct beetle assemblages. Potential loss of beetle family diversity in response to long-term red alder decline would compromise a wide range of ecosystem functions [129], and such impacts would likely be further exacerbated by habitat fragmentation. Our findings also emphasize the ecological importance of conserving dead wood, forest continuity, and structural heterogeneity to sustain family-level taxonomic and functional saproxylic beetle diversity in red alder ecosystems. We contend that assessing beetle diversity at the family level is useful for detecting broad trends in beetle communities in response to environmental variation while also simplifying the bycatch identification process. While some ecological associations are likely best captured at the species level, family level identification can foster efficient forest biodiversity assessment useful for forest management decisions and identifying ecological knowledge gaps, especially when analyses rely upon opportunistic or bycatch-derived data where financial, temporal, and expertise constraints often preclude identifications at fine taxonomic scales [8,9]. We also stress that trap bycatch represents a valuable resource for characterizing diversity trends in relation to site-specific factors. While bycatch is often omitted from data analyses owing to the aforementioned constraints or discarded without examination [130], incorporating beetle bycatch diversity analysis into long-term monitoring programs offers a cost-effective and resource-efficient way to inform conservation planning. Additionally, studying bycatch can reduce sampling waste and provide valuable data on the population statuses of rare, threatened, and endangered taxa, which should be prioritized in conservation efforts. We encourage researchers to preserve insect bycatch specimens and collaborate with museums and universities to make complete use of these informative resources.

## Supporting information

S1 Fig Photographs of representative specimens (1)–(45) from 45 beetle (Coleoptera) families captured across five study sites in the Lower Mainland, British Columbia, Canada, where family-level beetle bycatch diversity in red alder, *Alnus rubra*, stands was evaluated. Specimens are shown in dorsal (left), ventral (centre), and lateral (right) views. See Fig 2 for photographs of representative specimens of Salpingidae, Monotomidae, Nitidulidae, Staphylinidae, and Histeridae. Photographs by T. T. M. Yim.
(PDF)

S1 Table Summary of samples of beetle (Coleoptera) families captured as bycatch in Ultra-High Release ethanol-baited multiple-funnel traps across the five study sites in the Lower Mainland, British Columbia, Canada (*n* = 5 traps per site), where family-level beetle bycatch diversity in red alder, *Alnus rubra*, stands was evaluated. The total number of individuals recorded per family per site is reported. Site abbreviations: ARP = Aldergrove Regional Park, BL = Burnaby Lake Regional Park, MK = Malcolm Knapp Research Forest, PSP = Pacific Spirit Regional Park, TRP = Tynehead Regional Park.
(PDF)

S2 Table Summary of single-predictor negative binomial generalised linear models (GLMs) testing the effects of overall stand conditions, red alder status, and forest fragmentation metrics on the abundances of the five most common beetle bycatch families identified in this study that are saproxylic and contain scolytine predators across five study sites (*n* = 5 traps per site) dominated by red alder, *Alnus rubra*, in the Lower Mainland, British Columbia, Canada. All predictors were analyzed on their original scale. The *p*-value and standardised regression coefficient (*b*) are provided for each model. Grey-shaded cells indicate *p* < 0.05. Orange and blue cells indicate significant positive and negative relationships (*b*) between a diversity metric and a predictor, respectively. See Table 1 for predictor calculation details.
(DOCX)

## References

[1] Evans AV. Beetles of western North America. New Jersey: Princeton University Press; 2021.

[2] Evans AV. The lives of beetles: a natural history of Coleoptera. New Jersey: Princeton University Press; 2023.

[3] Ghannem S, Touaylia S, Boumaiza M. Beetles (Insecta: Coleoptera) as bioindicators of the assessment of environmental pollution. Hum Ecol Risk Assess. 2017;24(2):456–64. doi: 10.1080/10807039.2017.1385387

[4] McGeoch MA, Schroeder M, Ekbom B, Larsson S. Saproxylic beetle diversity in a managed boreal forest: importance of stand characteristics and forestry conservation measures. Divers Distrib. 2007;13(4):418–9. doi: 10.1111/j.1472-4642.2007.00350.x

[5] Grodsky SM, Hernandez RR, Campbell JW, Hinson KR, Keller O, Fritts SR, et al. Ground beetle (Coleoptera: Carabidae) response to harvest residue retention: implications for sustainable forest bioenergy production. Forests. 2019;11(1):48. doi: 10.3390/f11010048

[6] von Hoermann C, Jauch D, Kubotsch C, Reichel-Jung K, Steiger S, Ayasse M. Effects of abiotic environmental factors and land use on the diversity of carrion-visiting silphid beetles (Coleoptera: Silphidae): a large scale carrion study. PLoS One. 2018;13(5):e0196839. doi: 10.1371/journal.pone.0196839 29847551 PMC5976144

[7] Bevilacqua S, Anderson MJ, Ugland KI, Somerfield PJ, Terlizzi A. The use of taxonomic relationships among species in applied ecological research: baseline, steps forward and future challenges. Austral Ecol. 2021;46(6):950–64. doi: 10.1111/aec.13061

[8] Godfray HCJ, Knapp S, Wheeler QD. Taxonomic triage and the poverty of phylogeny. Philos Trans R Soc Lond B Biol Sci. 2004;359(1444):571–83. doi: 10.1098/rstb.2003.1452 15253345 PMC1693342

[9] Ebach MC. Taxonomy and the DNA barcoding enterprise. Zootaxa. 2011;2742(1):67–8. doi: 10.11646/zootaxa.2742.1.5

[10] Klimaszewski J, Webster RP, Langor DW, Brunke A, Davies A, Bourdon C. Aleocharine rove beetles of eastern Canada (Coleoptera, Staphylinidae, Aleocharinae): a glimpse of megadiversity. Springer Nature Switzerland AG; 2018.

[11] Johnson AJ, Atkinson TH, Beaver RA, Cognato AI, Jordal BH, Knížek M, et al. A comprehensive taxonomic checklist of bark and ambrosia beetle species (Curculionidae, Scolytinae). Zookeys. 2026;1271:1–285. doi: 10.3897/zookeys.1271.168928 41799683 PMC12966851

[12] Báldi A. Using higher taxa as surrogates of species richness: a study based on 3700 Coleoptera, Diptera, and Acari species in Central-Hungarian reserves. Basic Appl Ecol. 2003;4(6):589–93. doi: 10.1078/1439-1791-00193

[13] González E, Salvo A, Valladares G. Arthropods on plants in a fragmented Neotropical dry forest: a functional analysis of area loss and edge effects. Insect Sci. 2015;22(1):129–38. doi: 10.1111/1744-7917.12107 24446307

[14] Brennan KEC, Ashby L, Majer JD, Moir ML, Koch JM. Simplifying assessment of forest management practices for invertebrates: How effective are higher taxon and habitat surrogates for spiders following prescribed burning? For Ecol Manage. 2006;231(1–3):138–54. doi: 10.1016/j.foreco.2006.05.035

[15] Godoy BS, Faria APJ, Juen L, Sara L, Oliveira LG. Taxonomic sufficiency and effects of environmental and spatial drivers on aquatic insect community. Ecol Indic. 2019;107:105624. doi: 10.1016/j.ecolind.2019.105624

[16] Heino J, Soininen J. Are higher taxa adequate surrogates for species-level assemblage patterns and species richness in stream organisms? Biol Conserv. 2007;137(1):78–89. doi: 10.1016/j.biocon.2007.01.017

[17] Gaston KJ, Williams PH. Mapping the world’s species-the higher taxon approach. Biodivers Lett. 1993;1(1):2–8. doi: 10.2307/2999642

[18] Janssen P, Fortin D, Hébert C. Beetle diversity in a matrix of old-growth boreal forest: influence of habitat heterogeneity at multiple scales. Ecography. 2009;32(3):423–32. doi: 10.1111/j.1600-0587.2008.05671.x

[19] Seidler R. Patterns of biodiversity change in anthropogenically altered forests. Reference module in life sciences. Amsterdam: Elsevier; 2017. doi: 10.1016/B978-0-12-809633-8.02186-5

[20] Didham RK, Ghazoul J, Stork NE, Davis AJ. Insects in fragmented forests: a functional approach. Trends Ecol Evol. 1996;11(6):255–60. doi: 10.1016/0169-5347(96)20047-3 21237834

[21] Sánchez-Bayo F, Wyckhuys KAG. Worldwide decline of the entomofauna: A review of its drivers. Biol Conserv. 2019;232:8–27. doi: 10.1016/j.biocon.2019.01.020

[22] Didham RK, Hammond PM, Lawton JH, Eggleton P, Stork NE. Beetle species responses to tropical forest fragmentation. Ecol Monogr. 1998;68(3):295–323. doi: 10.1890/0012-9615(1998)068[0295:BSRTTF]2.0.CO;2

[23] Fujita A, Maeto K, Kagawa Y, Ito N. Effects of forest fragmentation on species richness and composition of ground beetle (Coleoptera: Carabidae and Brachinidae) in urban landscapes. Entomol Sci. 2008;11(1):39–48. doi: 10.1111/j.1479-8298.2007.00243.x

[24] Filgueiras BKC, Iannuzzi L, Leal IR. Habitat fragmentation alters the structure of dung beetle communities in the Atlantic Forest. Biol Conserv. 2011;144(1):362–9. doi: 10.1016/j.biocon.2010.09.013

[25] Wulder MA, White JC, Coops NC. Fragmentation regimes in Canada’s forests. Can Geogr. 2017;55(3):288–300. doi: 10.1111/j.1541-0064.2010.00335.x

[26] Lassau SA, Hochuli DF, Cassis G, Reid CAM. Effects of habitat complexity on forest beetle diversity: do functional groups respond consistently? Divers Distrib. 2005;11(1):73–82. doi: 10.1111/j.1366-9516.2005.00124.x

[27] Müller J, Bußler H, Kneib T. Saproxylic beetle assemblages related to silvicultural management intensity and stand structures in a beech forest in southern Germany. J Insect Conserv. 2008;12:107–24. doi: 10.1007/s10841-006-9065-2

[28] Rappa NJ, Staab M, Frey J, Winiger N. Multiple forest structural elements are needed to promote beetle biomass, diversity and abundance. For Ecosyst. 2022;9:100056. doi: 10.1016/j.fecs.2022.100056

[29] Harrington CA. Biology and ecology of red alder. In: Deal RL, Harrington CA, editors. Red alder: a state of knowledge. U.S. Department of Agriculture, Forest Service, Pacific Northwest Research Station; 2006. p. 21–44.

[30] Deal RL, Orlikowska EH, D’Amore DV, Hennon PE. Red alder-conifer stands in Alaska: an example of mixed species management to enhance structural and biological complexity. Forests. 2017;8(4):131. doi: 10.3390/f8040131

[31] Schultz ME, De Santo TL. Comparison of terrestrial invertebrate biomass and richness in young mixed red alder-conifer, young conifer, and old conifer stands of southeast Alaska. Northwest Sci. 2008;80(2):120–32.

[32] Perakis SS, Matkins JJ, Hibbs DE. N2-fixing red alder indirectly accelerates ecosystem nitrogen cycling. Ecosystems. 2012;15:1182–93. doi: 10.1007/s10021-012-9579-2

[33] Torrey JG. Nitrogen fixation by actinomycete-nodulated angiosperms. BioScience. 1978;28(9):586–92. doi: 10.2307/1307515

[34] Martín A, Etxebeste I, Pérez G, Álvarez G, Sánchez E, Pajares J. Modified pheromone traps help reduce bycatch of bark-beetle natural enemies. Agric For Entomol. 2013;15:86–97. doi: 10.1111/j.1461-9563.2012.00594.x

[35] Gugliuzzo A, Biedermann PHW, Carrillo D, Castrillo LA, Egonyu JP, Gallego D. Recent advances toward the sustainable management of invasive *Xylosandrus* ambrosia beetles. J Pest Sci. 2021;94:615–37. doi: 10.1007/s10340-021-01382-3

[36] Rudziński KJ, Sukovata L, Asztemborska M, Wróblewska A, Nestorowicz K, Szmigielski R. Developing an improved lure for attracting the pine sawyer beetle (*Monochamus galloprovincialis*) with reduced bycatch of predatory beetles. Agric For Entomol. 2024;26(4):433–44. doi: 10.1111/afe.12627

[37] Schroeder LM. Differences in responses to α-pinene and ethanol, and flight periods between the bark beetle predators *Thanasimus femoralis* and *T. formicarius* (Col.: Cleridae). For Ecol Manage. 2003;177(1–3):301–11. doi: 10.1016/S0378-1127(02)00441-3

[38] Allison JD, Redak RA. The impact of trap type and design features on survey and detection of bark and woodboring beetles and their associates: a review and meta-analysis. Annu Rev Entomol. 2017;62:127–46. doi: 10.1146/annurev-ento-010715-023516 27813665

[39] Montgomery ME, Wargo PM. Ethanol and other host-derived volatiles as attractants to beetles that bore into hardwoods. J Chem Ecol. 1983;9(2):181–90. doi: 10.1007/BF00988035 24407336

[40] Miller DR, Rabaglia RJ. Ethanol and (-)-alpha-Pinene: attractant kairomones for bark and ambrosia beetles in the southeastern US. J Chem Ecol. 2009;35(4):435–48. doi: 10.1007/s10886-009-9613-9 19294470

[41] Miller DR. Ethanol: dose-dependent flight responses of bark and woodboring beetles, and associated species of Coleoptera. Environ Entomol. 2025;54(3):467–79. doi: 10.1093/ee/nvaf032 40209107

[42] Ruchin AB, Egorov LV, Khapugin AA. Usage of fermental traps for the study of the species diversity of Coleoptera. Insects. 2021;12(5):407. doi: 10.3390/insects12050407 33946580 PMC8147233

[43] Touroult J, Witté I. Beer, wine, or fruit juice: which is best? A case study of bait efficiency to sample saproxylic beetles (Coleoptera) in an oak woodland. Coleopt Bull. 2020;74(4). doi: 10.1649/0010-065x-74.4.763

[44] Buchholz S, Kreuels M, Kronshage A, Terlutter H, Finch OD. Bycatches of ecological field studies: bothersome or valuable?. Methods Ecol Evol. 2011;2(1):99–102. doi: 10.1111/j.2041-210X.2010.00051.x

[45] Kühnholz S, Borden JH, Uzunovic A. Secondary ambrosia beetles in apparently healthy trees: adaptations, potential causes and suggested research. Integr Pest Manage Rev. 2001;6(3–4):209–19. doi: 10.1023/a:1025702930580

[46] Wertman DL. The evolution of bark beetle–fungus mutualisms: insights from a hardwood system. Ph.D. Thesis. The University of British Columbia; 2024.

[47] Wertman DL, Hamelin RC, Carroll AL. Special delivery: a hardwood‐killing bark beetle vectors its unusual symbiote among host trees. Ecosphere. 2025;16(5). doi: 10.1002/ecs2.70256

[48] Kenis M, Wermelinger B, Grégoire JC. Research on parasitoids and predators of Scolytidae – a review. In: Lieutier F, Day KR, Battisti A, Grégoire JC, Evans HF, editors. Bark and wood boring insects in living trees in Europe, a synthesis. Dordrecht: Springer; 2007. p. 237–90.

[49] Lindgren BS. A multiple funnel trap for scolytid beetles (Coleoptera). Can Entomol. 1983;115(3):299–302. doi: 10.4039/ent115299-3

[50] Rassati D, Faccoli M, Toffolo EP, Battisti A, Marini L. Improving the early detection of alien wood-boring beetles in ports and surrounding forests. J Appl Ecol. 2015;52(1):50–8. doi: 10.1111/1365-2664.12347

[51] Tadege M, Dupuis I I, Kuhlemeier C. Ethanolic fermentation: new functions for an old pathway. Trends Plant Sci. 1999;4(8):320–5. doi: 10.1016/s1360-1385(99)01450-8 10431222

[52] Miller DR, Duerr DA. Comparison of arboreal beetle catches in wet and dry collection cups with Lindgren multiple funnel traps. J Econ Entomol. 2008;101(1):107–13. doi: 10.1603/0022-0493(2008)101[107:coabci]2.0.co;2 18330123

[53] Hatch MH. The beetles of Pacific Northwest: part I: introduction and Adephaga. Washington: University of Washington Press; 1953.

[54] Hatch MH. The beetles of Pacific Northwest: part II: Staphyliniformia. Washington: University of Washington Press; 1957.

[55] Hatch MH. The beetles of Pacific Northwest: part III: Pselaphidae and Diversicornia I. Washington: University of Washington Press; 1962.

[56] Hatch MH. The beetles of Pacific Northwest: part IV: Macrodactyles, Palpicornes, and Heteromera. Washington: University of Washington Press; 1965.

[57] Hatch MH. The beetles of Pacific Northwest: part V: Rhipiceroidea, Sternoxi, Phytophaga, Rhynchophora, and Lamellicornia. Washington: University of Washington Press; 1971.

[58] Spencer Entomological Collection (UBCZ) from University of British Columbia. doi: 10.5886/r5d29ft9

[59] Natural Resources Canada, Canadian Forest Service. Canada’s National Forest Inventory ground sampling guidelines: specifications for ongoing measurement. Version 5.0. Pacific Forestry Centre. 2008 [cited 2025 Oct 19]. Available from: https://nfi.nfis.org/en

[60] Google Earth. Google Earth Pro. Version 7.3.6.10201 [software]. 2025 [cited 2025 Oct 19]. Available from: https://earth.google.com

[61] Evans MJ, Barton P, Niwa S, Soga M, Seibold S, Tsuchiya K, et al. Climate-driven divergent long-term trends of forest beetles in Japan. Ecol Lett. 2022;25(9):2009–21. doi: 10.1111/ele.14082 35904819

[62] Broadbent EN, Asner GP, Keller M, Knapp DE, Oliveira PJC, Silva JN. Forest fragmentation and edge effects from deforestation and selective logging in Brazilian Amazon. Biol Conserv. 2008;141(7):1745–57. doi: 10.1016/j.biocon.2008.04.024

[63] Noble CD, Gilroy JJ, Berenguer E, Vaz-de-Mello FZ, Peres CA. Many losers and few winners in dung beetle responses to Amazonian forest fragmentation. Biol Conserv. 2023;281:110024. doi: 10.1016/j.biocon.2023.110024

[64] Shannon CE. A mathematical theory of communication. Bell Syst Tech J. 1948;27(3):379–423. doi: 10.1002/j.1538-7305.1948.tb01338.x

[65] Whittaker RH. Evolution and measurement of species diversity. Taxon. 1972;21(2–3):213–51. doi: 10.2307/1218190

[66] Morris EK, Caruso T, Buscot F, Fischer M, Hancock C, Maier TS, et al. Choosing and using diversity indices: insights for ecological applications from the German Biodiversity Exploratories. Ecol Evol. 2014;4(18):3514–24. doi: 10.1002/ece3.1155 25478144 PMC4224527

[67] Levene H. Robust tests for equality of variances. In: Olkin I, editor. Contributions to probability and statistics. California: Standford University Press; 1960. p. 278–92.

[68] Shapiro SS, Wilk MB. An analysis of variance test for normality (complete samples). Biometrika. 1965;52(3–4):591–611. doi: 10.2307/2333709

[69] Fisher RA. Statistical methods for research workers. Edinburgh: Oliver and Boyd; 1925.

[70] Tukey JW. The philosophy of multiple comparisons. Stat Sci. 1991;6(1):100–16. doi: 10.1214/ss/1177011945

[71] Kruskal WH, Wallis WA. Use of ranks in one-criterion variance analysis. J Am Stat Assoc. 1952;47(260):583–621. doi: 10.2307/2280779

[72] Dunn OJ. Multiple comparisons among means. J Am Stat Assoc. 1961;56(293):52–64. doi: 10.2307/2282330

[73] Sellers KF, Borle S, Shmueli G. The COM-Poisson model for count data: a survey of methods and applications. Appl Stoch Model Bus Ind. 2012;28:104–16. doi: 10.1002/asmb.918

[74] Lindén A, Mäntyniemi S. Using the negative binomial distribution to model overdispersion in ecological count data. Ecology. 2011;92(7):1414–21. doi: 10.1890/10-1831.1 21870615

[75] Yim TTM, Zhou S, Wertman DL, Carroll AL. Data evaluating family-level beetle bycatch diversity in red alder ecosystems. Repository: Dryad. 2026 [cited 2025 Nov 5]. doi: 10.5061/dryad.31zcrjf0g

[76] Posit team. RStudio: integrated development environment for R. Posit Software, PBC. 2024 [cited 2025 Aug 1]. Available from: http://www.posit.co/

[77] R Core Team. R: a language and environment for statistical computing. R Foundation for Statistical Computing. 2024 [cited 2025 Aug 1]. Available from: https://www.R-project.org/

[78] Microsoft Corporation. Microsoft Excel. Version 16.95.1 [software]. 2025 [cited 2025 Oct 19]. Available from: https://www.microsoft.com/en-ca/microsoft-365/excel/

[79] Fox J, Wiesberg S, Price B, Adler D, Bates D, Baud-Bovy G. Car: companion to applied regression. Version 3.1-3. 2024 [cited 2025 Oct 19]. doi: 10.32614/CRAN.package.car

[80] Dinno A. dunn.test: Dunn’s test of multiple comparisons using rank sums. Version 1.3.6. 2024 [cited 2025 Oct 19]. doi: 10.32614/CRAN.package.dunn.test

[81] Brooks ME, Kristensen K, van Benthem KJ, Magnusson A, Berg CW, Nielsen A, et al. glmmTMB: generalized linear mixed models using template model builder. Version 1.1.11. 2025 [cited 2025 Oct 19]. doi: 10.32614/CRAN.package.glmmTMB

[82] Ripley B, Venables W, Bates DM, Hornik K, Gebhardt A, Firth D. MASS: support functions and datasets for Venables and Ripley’s MASS. Version 7.3-64. 2025 [cited 2025 Oct 19]. doi: 10.32614/CRAN.package.MASS

[83] Wickham H, François R, Henry L, Müller K, Vaughan D. dplyr: a grammar of data manipulation. Version 1.1.4 [R package]. Posit Software, PBC; 2023 [cited 2025 Oct 19]. doi: 10.32614/CRAN.package.dplyr

[84] Wickham H, Chang W, Henry L, Pedersen TL, Takahashi K, Wilke C. ggplot2: create elegant data visualisations using the grammar of graphics. Version 3.5.2. 2025 [cited 2025 Oct 19]. doi: 10.32614/CRAN.package.ggplot2

[85] Garnier S, Ross N, Rudis B, Sciaini M, Camargo AP, Scherer C. C viridis: colorblind-friendly color maps for R. Version 0.6.5 [R package]. 2024. doi: 10.32614/CRAN.package.viridis

[86] Grove SJ. Saproxylic insect ecology and the sustainable management of forests. Annu Rev Ecol Evol Syst. 2002;33:1–23. doi: 10.1146/annurev.ecolsys.33.010802.150507

[87] Lawrence JF, Ślipiński SA, Pollock DA, Escalona H. Salpingidae Leach, 1815. In: Kükenthal W, Leschen RAB, Beutel G, Lawrence JF, editors. Coleoptera, beetles. Volume 2: morphology and systematics (Elateroidea, Bostrichiformia, Cucujiformia partim). Berlin: De Gruyter; 2010. p. 722–9.

[88] Hammond HEJ, Chambers KLD. A review of the western Canadian and Alaskan species of Corticaria Marsham, 1802 (Coleoptera: Latridiidae): descriptions of new species and taxonomic notes on other North American species. Coleopt Bull. 2020;74(2):201–311. doi: 10.1649/0010-065X-74.2.201

[89] Bousquet Y. Monotomidae Laporte, 1840. In: Arnett RH, Thomas MC, Skelly PE, Frank JH, editors. American beetles, volume II: Polyphaga: Scarabaeoidea through Curuclionoidea. Florida: CRC Press; 2002. pp. 319–24.

[90] Sandström J, Bernes C, Junninen K, Lõhmus A, Macdonald E, Müller J, et al. Impacts of dead wood manipulation on the biodiversity of temperate and boreal forests. A systematic review. J Appl Ecol. 2019;56(7):1770–81. doi: 10.1111/1365-2664.13395

[91] Seibold S, Bässler C, Brandl R, Gossner MM, Thorn S, Ulyshen M. Experimental studies of dead-wood biodiversity — a review identifying global gaps in knowledge. Biol Conserv. 2015;191:139–49. doi: 10.1016/j.biocon.2015.06.006

[92] Seibold S, Bässler C, Brandl R, Büche B, Szallies A, Thorn S. Microclimate and habitat heterogeneity as the major drivers of beetle diversity in dead wood. J Appl Ecol. 2016;53(3):934–43. doi: 10.1111/1365-2664.12607

[93] Schiegg K. Effects of dead wood volume and connectivity on saproxylic insect species diversity. Écoscience. 2000;7(3):290–8. doi: 10.1080/11956860.2000.11682598

[94] Hottola J, Ovaskainen O, Hanski I. A unified measure of the number, volume and diversity of dead trees and the response of fungal communities. J Ecol. 2009;97(6):1320–8. doi: 10.1111/j.1365-2745.2009.01583.x

[95] Juutilainen K, Halme P, Kotiranta H, Mönkkönen M. Size matters in studies of dead wood and wood-inhabiting fungi. Fungal Ecol. 2011;4(5):342–9. doi: 10.1016/j.funeco.2011.05.004

[96] Seibold S, Müller J, Baldrian P, Cadotte MW, Štursová M, Biedermann PHW, et al. Fungi associated with beetles dispersing from dead wood – Let’s take the beetle bus!. Fungal Ecol. 2019;39:100–8. doi: 10.1016/j.funeco.2018.11.016

[97] Filipiak M, Sobczyk Ł, Weiner J. Fungal transformation of tree stumps into a suitable resource for *Xylophagous* beetles via changes in elemental ratios. Insects. 2016;7(2):13. doi: 10.3390/insects7020013

[98] Filipiak M. Nutrient dynamics in decomposing dead wood in the context of wood eater requirements: the ecological stoichiometry of saproxylophagous insects. In: Ulyshen M, editor. Saproxylic insects: diversity, ecology, and conservation. Zoological Monographs, vol 1. Cham: Springer; 2018. p. 429–69.

[99] Schigel DS. Fungus–beetle food web patterns in boreal forests. Russian Entomol J. 2011;20(1):141–50. doi: 10.15298/rusentj.20.2.05

[100] Økland B, Bakke A, Hågvar S, Kvamme T. What factors influence the diversity of saproxylic beetles? A multiscaled study from a spruce forest in southern Norway. Biodivers Conserv. 1996;5:75–100. doi: 10.1007/BF00056293

[101] Siitonen J. Microhabitats. In: Stokland JN, Siitonen J, Jonsson BG, editors. Biodiversity in dead wood. Cambridge: Cambridge University Press; 2012. p. 150–82.

[102] Lee MH, Hong K-J, Lee J-S, Lee S. Review of family Monotomidae Laporte, 1840 (Coleoptera: Cucujoidea) in Korea. J Asia-Pac Biodivers. 2020;13(4):539–44. doi: 10.1016/j.japb.2020.06.016

[103] Lee MH, Lee S, Leschen RAB, Lee S. Evolution of feeding habits of sap beetles (Coleoptera: Nitidulidae) and placement of Calonecrinae. Syst Entomol. 2020;45:911–23. doi: 10.1111/syen.12441

[104] Lipkow E, Betz O. Staphylinidae and fungi. Faun Ökol Mitt. 2005;8:383–411.

[105] Shortle WC, Dudzik KR. Wood decay in living and dead trees: a pictorial overview. U.S. Department of Agriculture, Forest Service, Northern Research Station; 2012.

[106] Reineke LH. Perfecting a stand-density index for even-aged forest. J Agric Res. 1933;46(7):627–38.

[107] Mbenoun M, Wingfield MJ, Misse AC, Roux J. Selective feeding behaviors illuminate patterns of sap beetle associations with ophiostomatoid fungi. Symbiosis. 2020;81(3):287–302. doi: 10.1007/s13199-020-00705-9

[108] Kovarik PW, Caterino MS. Histeridae Glyllenhal, 1808. In: Beutel RF, Leschen RAB, editors. Coleoptera, beetles. Volume 1: morphology and systematics (Archostemata, Adephaga, Myxophaga, Polyphaga partim. Berlin: De Gruyter; 2011. p. 190–222.

[109] Normann C, Tscharntke T, Scherber C. Interacting effects of forest stratum, edge and tree diversity on beetles. For Ecol Manage. 2016;361:421–31. doi: 10.1016/j.foreco.2015.11.002

[110] Sobek S, Steffan-Dewenter I, Scherber C, Tscharntke T. Spatiotemporal changes of beetle communities across a tree diversity gradient. Divers Distrib. 2009;15(4):660–70. doi: 10.1111/j.1472-4642.2009.00570.x

[111] Milberg P, Bergman K, Johansson H, Jansson N. Low host‐tree preferences among saproxylic beetles: a comparison of four deciduous species. Insect Conserv Divers. 2014;7(6):508–22. doi: 10.1111/icad.12074

[112] Klein BC. Effects of Forest Fragmentation on Dung and Carrion Beetle Communities in Central Amazonia. Ecology. 1989;70(6):1715–25. doi: 10.2307/1938106

[113] Koivula MJ, Vermeulen HJK. Highways and forest fragmentation – effects on carabid beetles (Coleoptera, Carabidae). Landsc Ecol. 2005;20:911–26. doi: 10.1007/s10980-005-7301-x

[114] Seibold S, Brandl R, Buse J, Hothorn T, Schmidl J, Thorn S, et al. Association of extinction risk of saproxylic beetles with ecological degradation of forests in Europe. Conserv Biol. 2015;29(2):382–90. doi: 10.1111/cobi.12427 25429849

[115] Ewers RM, Didham RK. Pervasive impact of large-scale edge effects on a beetle community. Proc Natl Acad Sci U S A. 2008;105(14):5426–9. doi: 10.1073/pnas.0800460105 18375751 PMC2291112

[116] Nichols E, Larsen T, Spectar S, Davis AL, Escobar F, Favila M, et al. Global dung beetle response to tropical forest modification and fragmentation: a quantitative literature review and meta-analysis. Biol Conserv. 2007;137(1):1–19. doi: 10.1016/j.biocon.2007.01.023

[117] Ewers RM, Didham RK. Confounding factors in the detection of species responses to habitat fragmentation. Biol Rev Camb Philos Soc. 2005;81(1):117–42. doi: 10.1017/S1464793105006949 16318651

[118] Bouget C, Brin A, Tellez D, Archaux F. Intraspecific variations in dispersal ability of saproxylic beetles in fragmented forest patches. Oecologia. 2015;177(3):911–20. doi: 10.1007/s00442-014-3162-9 25428787

[119] Magnago LFS, Edwards DP, Edwards FA, Magrach A, Martins SV, Laurance WF. Functional attributes change but functional richness is unchanged after fragmentation of Brazilian Atlantic forests. J Ecol. 2013;102(2):475–85. doi: 10.1111/1365-2745.12206

[120] McKinney M, Lockwood J. Biotic homogenization: a few winners replacing many losers in the next mass extinction. Trends Ecol Evol. 1999;14(11):450–3. doi: 10.1016/s0169-5347(99)01679-1 10511724

[121] Lettenmaier L, Bässler C, Decker O, Hagge J, Heibl C, Mamadashvili G, et al. 12 years of assembly patterns in saproxylic beetles suggest early decay wood as ephemeral resource patch. J Anim Ecol. 2026;95(2):282–95. doi: 10.1111/1365-2656.70183 41216996 PMC12868409

[122] Edmonds RL, Vogt DJ, Sandberg DH, Driver CH. Decomposition of Douglas-fir and red alder wood in clear-cuttings. Can J For Res. 1986;16(4):822–31. doi: 10.1139/x86-145

[123] Jäch MA, Balke M. Global diversity of water beetles (Coleoptera) in freshwater. Hydrobiologia. 2008;595:419–42. doi: 10.1007/s10750-007-9117-y

[124] Ikeda H, Kagaya T, Kubota K, Abe T. Evolutionary relationships among food habit, loss of flight, and reproductive traits: life-history evolution in the Silphinae (Coleoptera: Silphidae). Evolution. 2008;62(8):2065–79. doi: 10.1111/j.1558-5646.2008.00432.x 18507741

[125] Lövei GL, Sunderland KD. Ecology and behavior of ground beetles (Coleoptera: Carabidae). Annu Rev Entomol. 1996;41:231–56. doi: 10.1146/annurev.en.41.010196.001311 15012329

[126] Guerold F. Influence of taxonomic determination level on several community indices. Water Res. 2000;34(2):487–92. doi: 10.1016/S0043-1354(99)00165-7

[127] Ramírez-Hernández A, Micó E, Galante E. Temporal variation in saproxylic beetle assemblages in a Mediterranean ecosystem. J Insect Conserv. 2014;18(5):993–1007. doi: 10.1007/s10841-014-9706-9

[128] Kopr D, Šipoš J, Schlaghamerský J. Importance of stochastic assembly processes influencing beetle communities increases after logging. For Ecol Manage. 2023;545:121296. doi: 10.1016/j.foreco.2023.121296

[129] Ulyshen MD. Strengthening the case for saproxylic arthropod conservation: a call for ecosystem services research. Insect Conserv Divers. 2013;6(3):393–5. doi: 10.1111/j.1752-4598.2012.00220.x

[130] Petsopoulos D, Lunt DH, Bell JR, Kitson JJN, Collins L, Boonham N. Using network ecology to understand and mitigate long-term insect declines. Ecol Entomol. 2021;46(4):693–8. doi: 10.1111/een.13035

