# Supplementary material for: Trap tales: The influence of red alder stand conditions and forest fragmentation on family-level beetle bycatch diversity
Source: PLoS One. 2026 Jul 15;21(7):e0353780. doi: 10.1371/journal.pone.0353780 (PMC13372131; doi:10.1371/journal.pone.0353780)

(1) Agyrtidae: *Necrophilus hydrophiloides* Guérin-Méneville, 1834

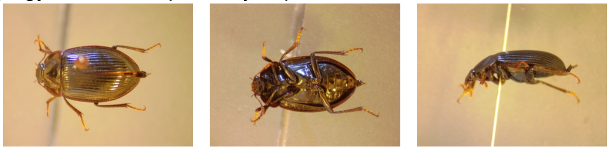

(2) Buprestidae: *Anthaxia* sp.

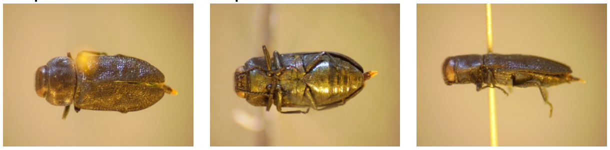

(3) Cantharidae: *Dichelotarsus piniphilus* (Eschscholtz, 1830)

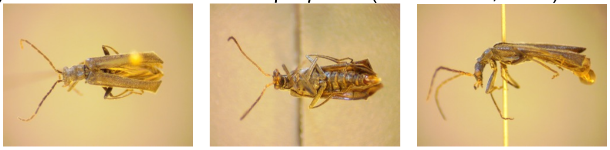

(4) Carabidae: *Anisodactylus* sp.

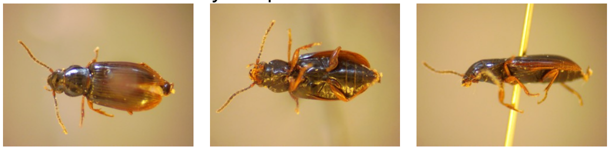

(5) Cerambycidae: *Plectura spinicauda* (Mannerheim, 1852)

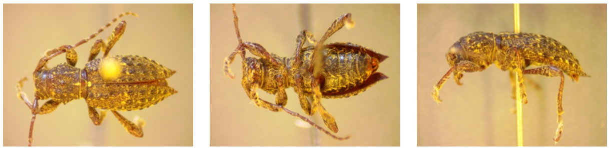

(6) Cerylonidae: *Cerylon unicolor* (Ziegler, 1845)

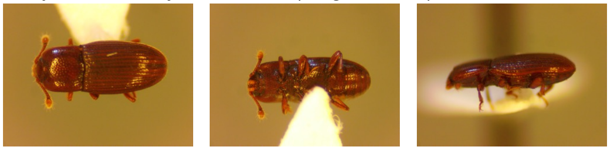

(7) Chrysomelidae: *Altica tombacina* (Mannerheim, 1853)

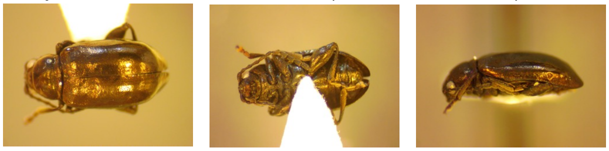

(8) Ciidae: *Cis* sp.

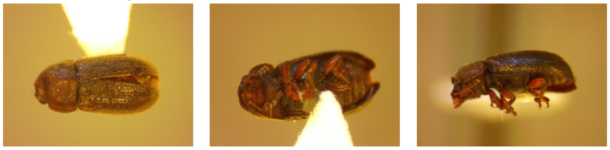

(9) Cleridae: *Thanasimus undatulus* (Say, 1835)

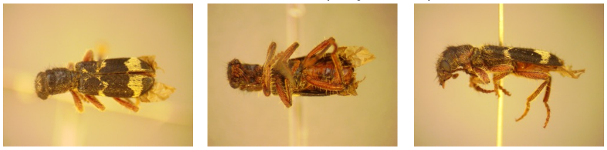

(10) Coccinellidae: *Psyllobora vigintimaculata* (Say, 1824)

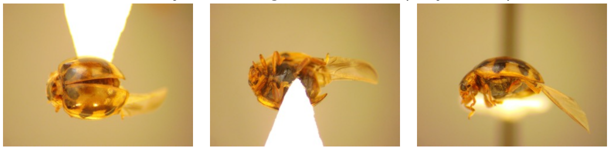

(11) Corylophidae: *Sericoderus lateralis* (Gyllenhal, 1827)

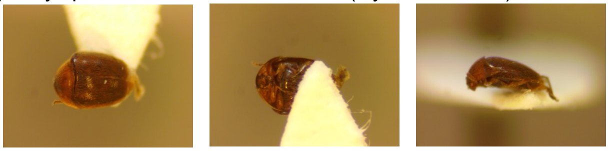

(12) Cryptophagidae: *Cryptophagus* sp.

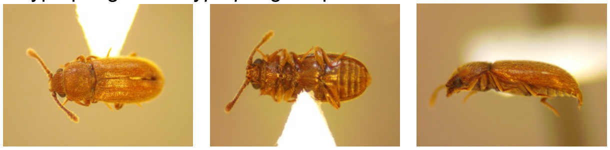

(13) Cucujidae: *Cucujus puniceus* Mannerheim, 1843

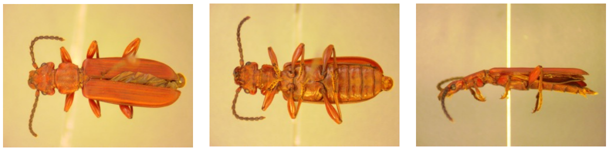

(14) Curculionidae: *Alniphagus aspericollis* (LeConte, 1876)

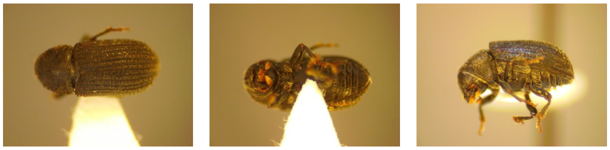

(15) Dermestidae: *Dermestes* sp.

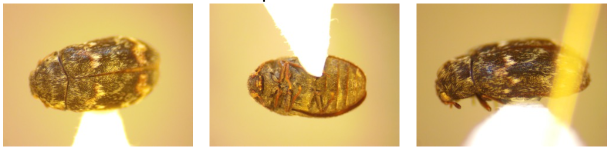

(16) Derodontidae: *Peltastica tuberculata* Mannerheim, 1852

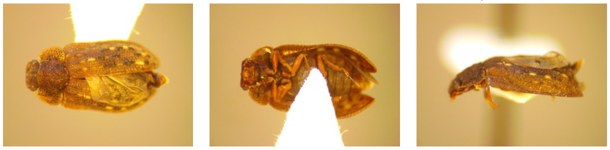

(17) Dysticidae: *Hydaticus aruspex* Clark, 1864

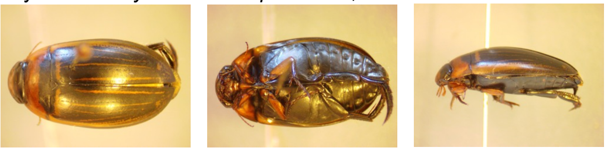

(18) Elateridae: *Selatosomus festivus* (LeConte, 1857)

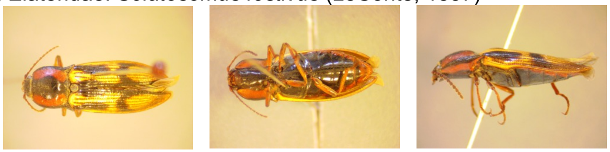

(19) Endomychidae: *Phymaphora pulchella* Newman, 1838

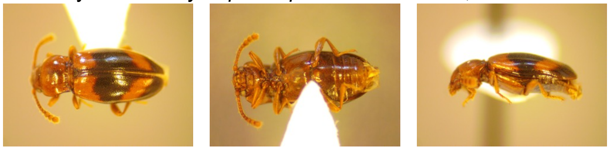

(20) Erotylidae: *Dacne californica* (Horn, 1870)

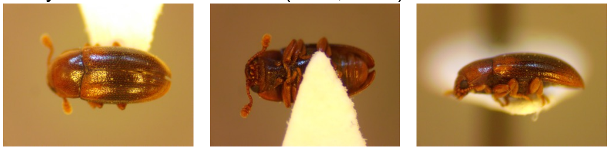

(21) Eucnemidae: *Melasis tsugae* Hopping, 1926

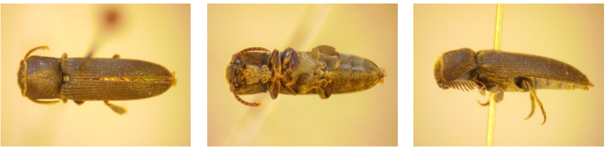

(22) Hydraenidae: *Ochthebius* sp.

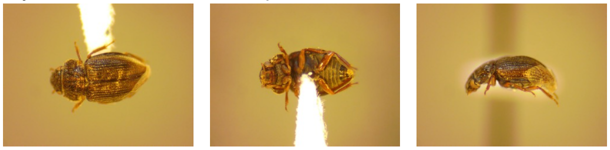

(23) Hydrophilidae: Unknown sp.

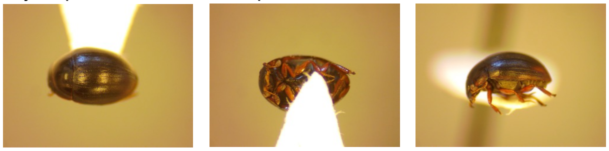

(24) Laemophloeidae: *Laemophloeus biguttatus* (Say, 1825)

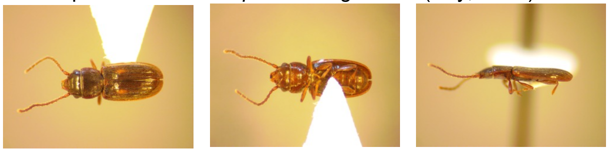

(25) Lampyridae: *Photinus* sp.

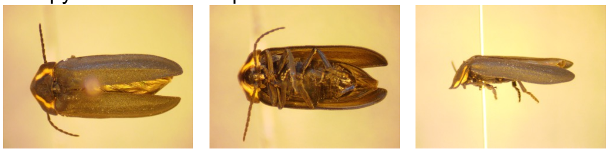

(26) Latridiidae: *Cartodere nodifer* (Westwood, 1839)

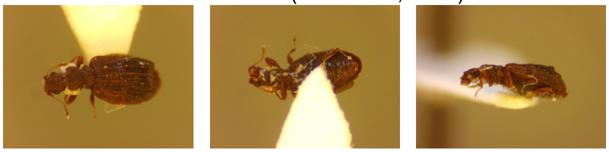

(27) Leiodidae: *Agathidium pulchrum* LeConte, 1853

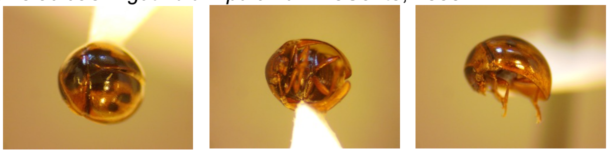

(28) Lucanidae: *Sinodendron rugosum* Mannerheim, 1843

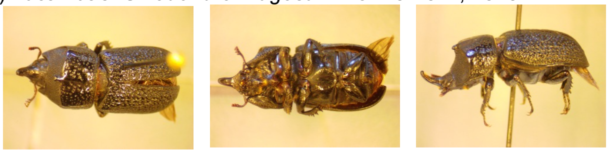

(29) Melandryidae: *Melandrya striata* Say, 1824

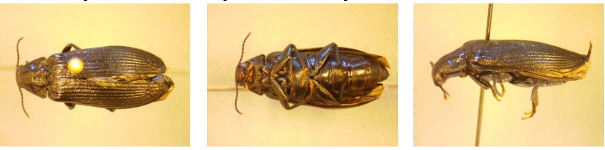

(30) Melyridae: *Dasytes plumbeus* (Müller, 1776)

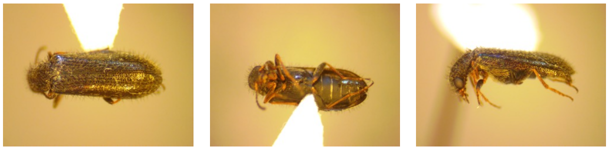

(31) Mordellidae: *Glipostenoda ambusta* (LeConte, 1862)

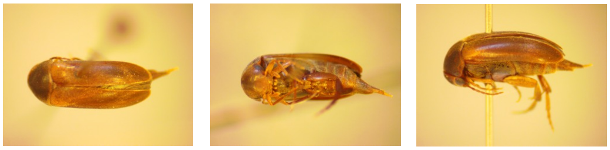

(32) Mycetophagidae: *Litargus* sp.

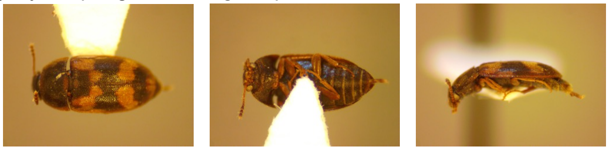

(33) Peltidae: *Peltis pippingskoeldi* (Mannerheim, 1852)

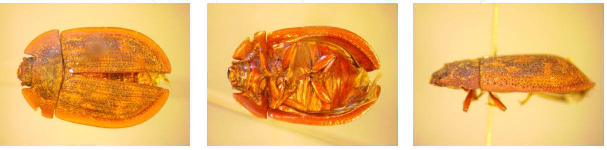

(34) Phalacridae: Unknown sp.

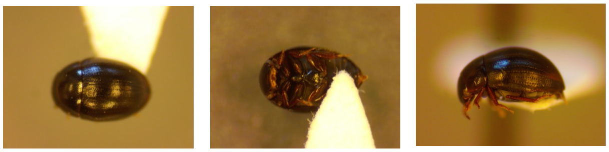

(35) Ptinidae: *Xestobium* sp.

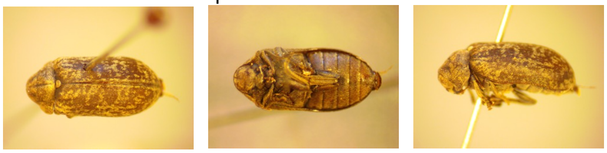

(36) Pyrochroidae: *Dendroides ephemeroides* (Mannerheim, 1852)

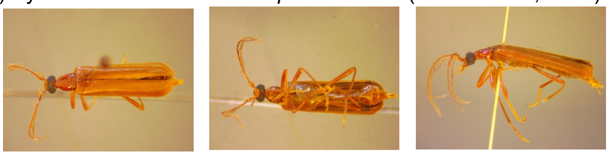

(37) Pythidae: *Sphalma quadricollis* Horn, 1872

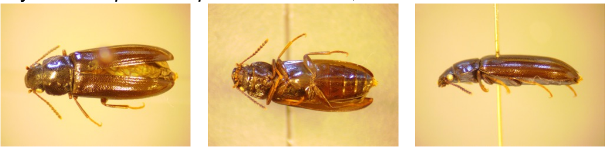

(38) Scarabaeidae: Unknown sp.

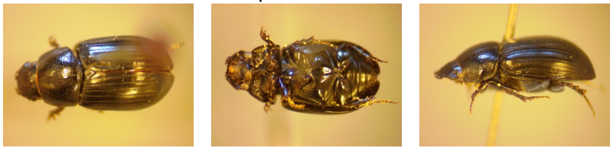

(39) Scirtidae: *Contacyphon* sp.

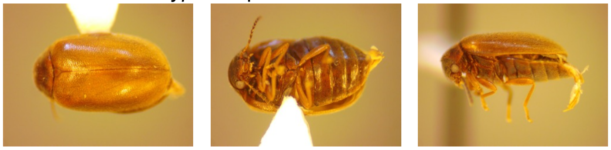

(40) Scraptiidae: *Anaspis* sp.

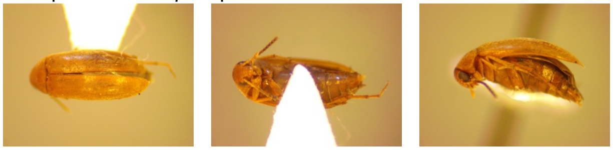

(41) Silphidae: *Nicrophorus defodiens* (Mannerheim, 1846)

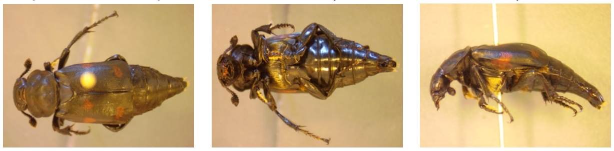

(42) Silvanidae: *Silvanus bidentatus* (Fabricius, 1792)

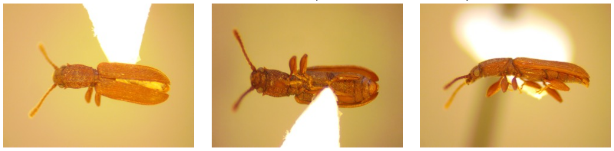

(43) Tenebrionidae: Unknown sp.

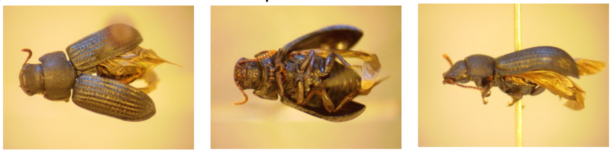

(44) Throscidae: *Trixagus* sp.

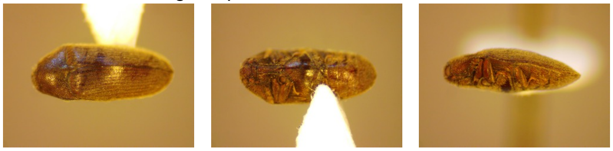

(45) Zopheridae: *Namunaria pacifica* (Horn, 1878)

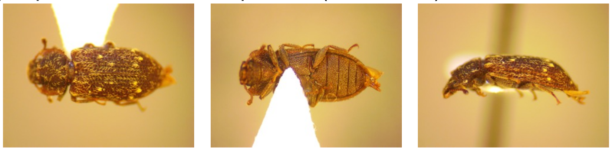

Supplement: S1 Fig — Specimens are shown in dorsal (left), ventral (centre), and lateral (right) views. See Fig 2 for photographs of representative specimens of Salpingidae, Monotomidae, Nitidulidae, Staphylinidae, and Histeridae. Photographs by T. T. M. Yim. (PDF) [file pone.0353780.s001.pdf]
