# Supplementary material for: Trap tales: The influence of red alder stand conditions and forest fragmentation on family-level beetle bycatch diversity
Source: PLoS One. 2026 Jul 15;21(7):e0353780. doi: 10.1371/journal.pone.0353780 (PMC13372131; doi:10.1371/journal.pone.0353780)
Supplement: S1 Table — The total number of individuals recorded per family per site is reported. Site abbreviations: ARP = Aldergrove Regional Park, BL = Burnaby Lake Regional Park, MK = Malcolm Knapp Research Forest, PSP = Pacific Spirit Regional Park, TRP = Tynehead Regional Park. (PDF) [file pone.0353780.s002.pdf]

Table S1. **Summary of samples of beetle (Coleoptera) families captured as bycatch in Ultra-High Release (UHR) ethanol-baited multiple-funnel traps across the five red alder-dominated study sites in the Lower Mainland of British Columbia, Canada (five traps per site).** The total number of individuals recorded per family per site is reported. Site abbreviations: ARP = Aldergrove Regional Park, BL = Burnaby Lake, MK = Malcolm Knapp Research Forest, PSP = Pacific Spirit Regional Park, TRP = Tynehead Regional Park.

	**Site**				
**Beetle family**	**ARP**	**BL**	**MK**	**PSP**	**TRP**
Agyrtidae	1	1	0	0	0
Buprestidae	0	0	0	0	1
Cantharidae	0	0	0	0	1
Carabidae	2	2	0	1	0
Cerambycidae	3	2	6	7	1
Cerylonidae	0	1	2	2	1
Chrysomelidae	1	4	0	0	0
Ciidae	5	8	29	18	12
Cleridae	10	0	11	0	10
Coccinellidae	0	2	0	2	0
Corylophidae	2	1	4	3	2
Cryptophagidae	2	7	3	35	6
Cucujidae	2	0	13	3	0
Curculionidae[1]	20	33	93	37	20
Dermestidae	0	1	0	0	1
Derodontidae	4	11	161	63	18
Dystiscidae	0	1	0	0	0
Elateridae	15	7	18	10	12
Endomychidae	0	0	1	0	1
Erotylidae	18	21	95	15	9
Eucnemidae	0	2	17	0	0
Histeridae	5	4	137	55	5
Hydraenidae	0	0	0	1	0
Hydrophilidae	0	2	0	0	0
Laemophloeidae	5	6	5	0	2
Lampyridae	1	1	1	0	0
Latridiidae	397	374	582	243	369

Leiodidae	6	1	8	7	1
Lucanidae	5	5	12	41	13
Melandryidae	0	0	10	1	2
Melyridae	0	95	1	3	84
Monotomidae	114	287	149	244	50
Mordellidae	1	2	0	0	0
Mycetophagidae	42	594	19	82	77
Nitidulidae	32	50	198	134	25
Peltidae	0	2	2	0	0
Phalacridae	0	1	1	0	0
Ptinidae	4	8	7	12	3
Pyrochroidae	1	1	5	1	0
Pythidae	0	0	1	0	0
Salpingidae	306	391	474	396	517
Scarabaeidae	3	0	0	1	1
Scirtidae	0	53	2	1	0
Scraptiidae	2	8	7	5	0
Silphidae	1	8	1	0	1
Silvanidae	2	7	17	1	2
Staphylinidae	72	51	117	47	45
Tenebrionidae	1	2	1	5	1
Throscidae	1	5	1	1	1
Zopheridae	5	15	55	19	2
**Total**	1091	2077	2266	1496	1296

[1]Excluding ambrosia beetles (Curculionidae: Scolytinae). Bark beetles (Scolytinae) and other members of the family Curculionidae, except ambrosia beetles, were included in the analyses.
