# Supplementary material for: Trap tales: The influence of red alder stand conditions and forest fragmentation on family-level beetle bycatch diversity
Source: PLoS One. 2026 Jul 15;21(7):e0353780. doi: 10.1371/journal.pone.0353780 (PMC13372131; doi:10.1371/journal.pone.0353780)
Supplement: S2 Table — All predictors were analyzed on their original scale. The p-value and standardised regression coefficient (b) are provided for each model. Grey-shaded cells indicate p < 0.05. Orange and blue cells indicate significant positive and negative relationships (b) between a diversity metric and a predictor, respectively. See Table 1 for predictor calculation details. (DOCX) [file pone.0353780.s003.docx]

**Table S2**. **Summary of single-predictor negative binomial generalised linear models (GLM) testing the effects of stand condition and forest fragmentation metrics on the abundances of the five most common beetle families identified in this study that are saproxylic and contain scolytine predators.** All predictors were analyzed on their original scale. The *p*-value and standardised regression coefficient (*b*) are provided for each model. Grey-shaded cells indicate *p* < 0.05. Orange and blue cells indicate significant positive and negative relationships (*b*) between a diversity metric and a predictor, respectively. See Table 1 for predictor calculation details.

|  | **Salpingidae** | | **Monotomidae** | | **Nitidulidae** | | **Staphylinidae** | | **Histeridae** | |
| --- | --- | --- | --- | --- | --- | --- | --- | --- | --- | --- |
| **Predictor** | *p* | *b* | *p* | *b* | *p* | *b* | *p* | *b* | *p* | *b* |
| **Overall stand condition** |  |  |  |  |  |  |  |  |  |  |
| Total scolytine abundance | 0.0452 | 1.8090 × 10^-5^ | 0.5150 | 9.9710 × 10^-6^ | 0.0055 | 5.0920 × 10^-5^ | 0.9900 | 1.5080 × 10^-7^ | 0.0040 | 0.0001 |
| Tree density^1,3^ | 0.9320 | -2.7940 × 10^-5^ | < 0.001 | 0.0015 | < 0.001 | 0.0029 | 0.2200 | 0.0005 | < 0.001 | 0.0051 |
| Living tree density | 0.7480 | -0.0002 | 0.6710 | 0.0003 | 0.0054 | 0.0025 | 0.9720 | 2.1930 × 10^-5^ | 0.0056 | 0.0012 |
| Mean tree DBH^1,2,3^ | 0.7390 | 0.0041 | < 0.001 | -0.0763 | < 0.001 | -0.1037 | 0.0233 | -0.0324 | < 0.001 | -0.1684 |
| Mean living tree DBH^2^ | 0.7750 | 0.0034 | < 0.001 | -0.0724 | < 0.001 | -0.0998 | 0.0148 | -0.0335 | < 0.001 | -0.1621 |
| Tree species richness^1,3^ | 0.0637 | -0.0670 | 0.0026 | 0.2211 | 0.4410 | 0.0598 | 0.9080 | -0.0055 | 0.6365 | -0.0676 |
| Tree species diversity^1,3^ | 0.0675 | -0.2510 | < 0.001 | 0.8908 | 0.2521 | 0.3930 | 0.9400 | 0.0583 | 0.5640 | -0.3127 |
| **Red alder-specific stand condition** |  |  |  |  |  |  |  |  |  |  |
| Red alder density^1,3^ | 0.2880 | 0.0005 | 0.4620 | -0.0006 | < 0.001 | 0.0033 | 0.0176 | 0.0013 | < 0.001 | 0.0077 |
| Dead red alder density^3^ | 0.6020 | 0.0002 | 0.4750 | 0.0005 | < 0.001 | 0.0039 | 0.0175 | 0.0012 | < 0.001 | 0.0075 |
| Living red alder density | 0.3180 | 0.0011 | < 0.001 | -0.0080 | 0.0023 | -0.0065 | 0.6740 | -0.0006 | 0.0210 | -0.0097 |
| Mean red alder DBH^1,2,3^ | 0.6940 | -0.0040 | 0.3100 | -0.0163 | < 0.001 | -0.0885 | 0.0424 | -0.0236 | < 0.001 | -0.1715 |
| Mean dead red alder DBH^2,3^ | 0.7350 | 0.0032 | 0.2980 | 0.0155 | < 0.001 | 0.0808 | 0.1200 | 0.0175 | < 0.001 | 0.1608 |
| Mean living red alder DBH^2^ | 0.7350 | -0.0035 | 0.2690 | -0.0179 | < 0.001 | -0.0892 | 0.0735 | -0.0215 | < 0.001 | -0.1739 |
| **Forest fragmentation metric** |  |  |  |  |  |  |  |  |  |  |
| Forest area | 0.2470 | 0.0052 | 0.0371 | -0.0142 | 0.0389 | 0.0176 | 0.0021 | 0.0146 | 0.0071 | 0.0401 |
| Number of forest patches | 0.6330 | 0.0055 | < 0.001 | -0.0721 | < 0.001 | -0.0821 | 0.9740 | 0.0005 | 0.0015 | -0.1296 |
| Mean forest patch area | 0.9120 | -4.244 × 10^-8^ | 0.1370 | 8.7330 × 10^-7^ | 0.0020 | 2.0950 × 10^-6^ | 0.5140 | -3.1370 × 10^-7^ | 0.0102 | 3.4460 × 10^-6^ |
| Mean edge-to-area ratio | 0.9700 | -0.1258 | 0.0030 | -13.7310 | < 0.001 | -24.0052 | 0.7710 | 1.1952 | < 0.001 | -43.5728 |

^1^Inclusive of alive and standing dead trees.

^2^DBH refers to diameter at breast height, measured at 1.3 m upslope.

^3^Dead trees included standing dead trees only; all observed standing dead trees in the 11.28 m-radius plots were red alders.
