# Supplementary material for: Trap tales: The influence of red alder stand conditions and forest fragmentation on family-level beetle bycatch diversity
Source: PLoS One. 2026 Jul 15;21(7):e0353780. doi: 10.1371/journal.pone.0353780 (PMC13372131; doi:10.1371/journal.pone.0353780)
Supplement: Attachment — Submitted filename: Yim et al_2026_Response to Reviewers.docx [file pone.0353780.s005.docx]

25 May 2026

Dear Dr. Frank Koch, Academic Editor, *PLOS One*,

We, Ting Ting Michelle Yim, Sichen Zhou, Debra Wertman, and Allan Carroll, the authors of “Trap tales: The influence of red alder stand conditions and forest fragmentation on family-level beetle bycatch diversity” (PONE-D-26-07315), thank the editor and reviewers for their constructive feedback on our manuscript. This letter addresses the Academic Editor’s and reviewers’ comments, indicating where and how the suggestions were incorporated to greatly improve our manuscript.

Editor and Reviewer comments are provided in italics with our responses following each in blue plain text. Unless otherwise stated, line numbers referenced by the reviewers correspond to the submitted manuscript, while the line numbers we referenced correspond to the revised manuscript (clean version).

**Academic Editor Comments**

*1. I agree with you (as do all the reviewers) about the information value of insect trap bycatch, and so I applaud you for undertaking this effort. I think the main criticism that emerges from the review comments is that you need to provide a fuller account of the methodological limitations of the study in your Discussion. You should also reexamine your conclusions, some of which should probably be tempered due to these limitations. Additionally, some aspects of your manuscript would benefit from clarification -- please look to the specific comments for guidance in that regard. Again, the reviewers and I see the merits of your manuscript, and because it is well-written and organized overall, I would be happy to reconsider it if you make the necessary changes.*

We thank the editor and reviewers for recognizing the significance of bycatch in ecological research and for their constructive feedback. Our response here is a summary of the major comments we have addressed.

Overall, we have added a clearer, more tempered account of our methodological limitations in the Discussion, including a dedicated limitations paragraph. We present the following summary of the specific methodological limitations raised by the Academic Editor and reviewers (please see our responses to the specific comments below for additional details):

(1) Sampling bias towards saproxylic taxa: While we had initially commented on this issue in the Discussion (original manuscript L511–517, revised L676–683), we have now added a cautionary statement on data interpretation related to this inherent sampling bias (L547–549).
(2) Coarse taxonomic resolution: We retain our initial perspective that family-level diversity metrics are useful and informative for analyses of ecosystem structure and function (original manuscript L645–648, revised L711–714), but have now expanded on our choice of family-level resolution for this study in the Introduction (original manuscript L54–58, revised L55–65) and further discuss our rationale in the Discussion (L714–719).

While we had initially indicated that the family-level resolution implemented in our study may hinder underlying species turnover processes in relation to fragmentation (original manuscript L608–616, revised L646–654), we have now added text clarifying that family-level resolution may inflate perceived diversity indices (L683–685) and that finer resolutions would be suitable for inferring within-family compositional shifts (L654–656).

(3) Small sample size (traps and plots within sites) and related statistical constraints: In the revised Discussion, we have explicitly mentioned that the relatively small number of sampling sites (5) and traps (5 per site), and the fixed-area plot configuration, may have incompletely captured landscape diversity and constrained our statistical analyses, including preventing the use of multivariable models (L689–695).

We had initially stated that our measurement of dead red alder availability represents a conservative estimate due to the limited number of sites (2) where dead red alders were observed in fixed-area plots (original manuscript L208–211, revised L225–228), and noted non-independence and inflated risk of Type I errors in the Methods (original manuscript L288–290, revised L307–309). We have now expanded on these limitations in the Discussion (L689–698). We have clarified the reasons for adopting bivariate models (*i.e*., models with one predictor and one response variable) in the Methods (L311–313) and Discussion (L692–695). We also now specify in the Methods that no predictor ranking or model comparison were conducted (L314) and indicate in the Discussion that results should therefore be interpreted as exploratory (L697–698).

(4) Temporal limitation: We have explicitly stated that our study represents a temporal snapshot and does not indicate interannual variability (L699–701).
(5) Fragmentation metrics: We have clarified that the use of relatively simple fragmentation metrics limits causal interpretations (L656–660).

*2. L17: insert comma after "diversity"*

We have made this edit (L17).

*3. L81: should be "promotes" instead of "promote"*

We have made this edit (L88).

*4. L202-203 ("...and dead if foliage was absent for deciduous trees ..."): This implies that the stands were inventoried in leaf-on conditions. Was that the case? I would indicate in the text.*

All stands were inventoried in leaf-on conditions in early to mid-August 2024. We have indicated this in the revised manuscript (L209–210).

*5. L208-211: So, in retrospect, would a different plot configuration have been better? For example, three smaller fixed-area plots at each location. Or would the result have been essentially the same? This is primarily a point for discussion (i.e., plot configuration matters, perhaps especially with small sample sizes).*

We thank the Academic Editor for this thoughtful comment. The use of the 11.28 m-radius fixed-area plot configuration is a conventional approach used by the Canadian National Forest Inventory Program (cited on original manuscript L193, revised L209) and provincial forest resource monitoring programs in British Columbia (e.g., Provincial Change Monitoring Inventory, ground-truthing for Vegetation Resources Inventory). It is applied to estimate forest stand conditions of forests dominated by large trees defined by diameter at breast height (DBH) ≥ 9 cm at a broad geographical scale. In our study, we applied a slightly lower threshold (DBH ≥ 8 cm) intended to accommodate smaller red alder trees, following Wertman *et al.* (2025), which we have now cited on L214.

However, we acknowledge that the plot configuration and a single plot per site could not fully capture the variability present within declining red alder stands, and thus the presence of standing dead red alders were missed at three sites. We have revised the Discussion to address this limitation (L551–553, 689–692).

*6. L289-292: I agree that it makes sense to prioritize minimizing false negatives.*

We thank the editor for acknowledging our analytical approach.

*7. L345: use "spp." instead of "sp."*

We have made this edit (L373).

*8. L349: replace "comprising" with "comprised of"*

We have made this edit (L377).

*9. L376-378: Did the five sites differ noticeably in the main drivers of fragmentation? For instance, MK was the most remote but had a moderate level of forestry-related fragmentation. Were any of other sites similar? (Another point for discussion.)*

Yes, the five sites differed with respect to main drivers of forest fragmentation. All sites, except MK, do not have forestry activities as they are managed for conservation, education, and recreation under the Metro Vancouver Regional Parks Plan (https://metrovancouver.org/services/regional-parks/regional-parks-plan), rather than for timber harvest and other forestry activities. We have noted that fragmentation drivers were identified from Google Earth satellite imagery in the Methods (L247–248), clarified the site-specific fragmentation drivers in the Results (L402–405, 407–409, 414), and mentioned them briefly in relation to beetle diversity and abundances in the Discussion (L620–628, 630–631).

*10. L385-387: See previous comment. Did BL have any forestry activity?*

BL does not have any forestry activity (L414), although it has a comparable number of forest patches and mean edge-to-area ratio as MK where fragmentation is primarily driven by forestry. We have now clarified the fragmentation drivers and indicate reasons for its lowest total forest cover in the Results (L404–405, 407–409).

*11. L388-392: I do worry somewhat that five sites are not sufficiently representative of the landscape diversity. (Again, a point for discussion.)*

Although our sample size was limited to five sites, these locations were carefully selected to capture a range of forest landscapes spanning naturalized to suburban/urban conditions in the Lower Mainland of British Columbia. However, we acknowledge that our sample size may not completely represent the full spectrum of the landscape diversity of red alder forests that beetles inhabit and may limit the generalizability of our conclusions. We have revised the Discussion to address this limitation (L689–692).

*12. L459-465: I'm not sure we can put too much stock in these results. 60% of the traps were in sites where there were no dead red alders recorded in the forest plot, so they're all represented as zeros even if there was some variation in dead red alder presence within the larger area. Again, in retrospect, would you have been better served by a different inventory plot configuration?*

Please see our response to Academic Editor Comment #5 for the rationale for the plot configuration used and how this limitation was incorporated in the Discussion. We have tempered the language of our conclusions regarding dead red alder availability in the Discussion (L551–552, 556, 558, 560, 565, 570–571, 575).

*13. L478-479: I think we can put more stock in these results, given the between-site variation.*

We agree with the Academic Editor on this point and have expanded upon our conclusions in the Discussion regarding between-site variation in fragmentation drivers in relation to diversity and abundances (L620–631).

**Reviewer 1 Comments**

*1. Redo the abstract. Specify exact numbers, not abstract words.*

We have revised the Abstract to include additional quantitative information (L25–28), more precise conclusions (L33–36), and a cautionary note regarding the limitations of the study (L38–39).

*2. The introduction lacks information about beetle baits based on beer, sugar, ethanol, and other agents. See such publications (https://doi.org/10.1134/S0013873824010056; https://doi.org/10.1007/s10841-017-9965-3; https://doi.org/10.1649/0010-065X-74.4.763; https://doi.org/10.3390/insects15060420) and give their analysis separately.*

We thank Reviewer 1 for this suggestion. The use of semiochemical baits in forest beetle pest monitoring is already described in the Introduction (original manuscript L100–102, revised L107–19). We have further introduced ethanol (L110–112), a kairomone widely used in forest scolytine monitoring (including in this study), as well as beer, wine, and sugar (L112–115), citing two of the recommended references (L114), in the revised Introduction.

*3. Why weren't the beetles identified before the species?*

We have interpreted this comment as “*Why weren’t the beetles identified to species?*” We implemented family-level identification as a practical and efficient method to assess broad beetle community patterns to inform management strategies, which is especially useful when expertise, time, and financial limitations are present in ecological studies. Our results showed that family-level resolution was indeed informative for detecting important ecological associations of the saproxylic beetle community in red alder forests and hence valuable for conservation and management applications.

We had initially outlined the rationale for using family-level identification in the original manuscript (L54–58) but have now expanded on and clarified this rationale in the revised Introduction (L55–65) and Discussion (L711–719), respectively. We have also explicitly addressed the limitations of this approach in the Discussion (L683–689). We have added additional supporting references to the Introduction and Discussion to support these revisions (L56–59, 64–65, 685, 719).

*4. Why do I need Figure 2? It shows nothing and is of very poor quality.*

Figure 2 presents five beetle families, each containing scolytine predators, that were evaluated for their responses to stand conditions and forest fragmentation. This figure is intended to provide a visual reference to support interpretation of the results for readers who work in forest, biodiversity, and conservation management who may not be taxonomic specialists in these beetle families. For this reason, we have retained Figure 2 in the revised manuscript and ensured that it meets the journal requirements.

*5. Make a conclusion*

The final paragraph of the Discussion presents our conclusions (L702–728). It summarizes our findings and demonstrates the practical implications of family-level identification and the ecological significance of bycatch. If the journal prefers, we can move this paragraph to a separate Conclusion section, but we do like how this information currently flows from the main content of the Discussion.

**Reviewer 2 Comments**

*1. I enjoyed reading the manuscript about beetle family diversity in red alder ecosystems. It is well written and structured. Methods and analyses seem very solid.*

*I appreciate the authors’ use of bycatch from existing insect traps for ecological analysis. I agree that bycatch deserves ethical consideration and can be a valuable and cost effective source of data for many ecological research questions.*

We thank Reviewer 2 for their positive feedback on our manuscript.

*2. I also recognize the limitations of this sampling method. Because these traps are designed mainly to catch flying, terrestrial insects, some beetle families are likely underrepresented or missed entirely. For example, mostly ground dwelling or flightless groups, such as Carabidae, are unlikely to be sampled well using this approach.*

*I therefore suggest adding a clearly defined paragraph or section that explicitly discusses the limitations of the study and of this sampling method. Although these limitations are mentioned briefly, especially in the first paragraph of the Discussion, I think they should be stated more clearly and in a more structured way. This would improve transparency and help readers better understand how broadly the results can be interpreted.*

We thank Reviewer 2 for this suggestion. While we had initially commented on the issue of sampling bias in the Discussion (original manuscript L511–517, revised L676–683), we have now consolidated this into a dedicated limitations paragraph (L675–701) and added a cautionary statement noting that data interpretation is limited to flying terrestrial saproxylic beetles (L547–549). Please see our response to the Academic Editor Comment #1 for details on how each limitation was addressed in the revised manuscript.

*3. Regarding the first paragraph of the Discussion, I suggest revising it substantially. Much of the information presented there seems more appropriate for the Results section. For example, the Discussion repeats which beetle families were captured as bycatch, even though this information is already presented in the Results and Table S2. The Results section already reports the total number of beetles and families captured, as well as the most abundant families. The Discussion would be stronger if this repetition were reduced and the focus shifted toward interpretation, including methodological limitations such as possible taxonomic biases in sampling.*

We thank Reviewer 2 for this observation. We have moved the information on captured beetle families from the first paragraph of the Discussion (original manuscript L501–506, 508–511) to the Results (L353–357, 360–364). We have removed Laemophloeidae and Silvanidae as examples of predatory families (original manuscript L509) as their predatory status is described as potential rather than confirmed in Kenis et al. (2007) (cited on original manuscript L509, revised L364). We have also removed the citation focusing exclusively on Cleridae as it was redundant (cited on original manuscript L509), given that Kenis et al. (2007) already provides a comprehensive overview of major predatory families of scolytines. Further, we have removed L507–508 of the original manuscript regarding captures of xylophagous families, along with two associated references, as this information is already provided in Table S2. Please see our response to Reviewer 2 Comment #2 above and to the Academic Editor Comment #1 for further details on how we have addressed methodological limitations including sampling bias towards saproxylic taxa in the revised Discussion.

*4. I also have a question about the field measurements. Were all stand level metrics collected by the same researcher? If not, how was observer bias addressed? This is especially relevant for measurements such as DBH, where small differences among observers could affect the results. Clarifying this would increase confidence in the reliability of the field data.*

All stand measurements were collected by the same field crew during the same time period (early to mid-August 2024) to ensure consistency and minimize observer bias.

*5. The authors use single predictor models to assess the relationship between stand conditions, fragmentation metrics and beetle diversity. While this approach is useful for exploratory analyses, it does not allow assessment of the relative importance of predictors or their effects after accounting for correlations among variables.*

*Could the authors clarify why multivariate models were not used, for example after checking collinearity among predictors? Including multiple predictors in a single model (with standardized coefficients) would allow direct comparison of effect sizes and help identify which variables have the strongest associations with richness or diversity.*

*If the intention is not to rank predictors, this limitation should be stated more explicitly. It would also be helpful to clarify whether single predictor models were compared (e.g. using AICc), and what conclusions can reasonably be drawn from such comparisons.*

We have interpreted this comment as referring to “*multivariable*” models (*i.e.*, models that include more than one predictor), as opposed to “*multivariate*” models (*i.e.*, models that have more than one response variable). We agree that multivariable models would be valuable for evaluating the relative importance of predictors; however, we used bivariate models (*i.e.,* models that have one predictor and one response variable) after careful consideration of our study design and objectives. Our primary aim was exploratory, *i.e.*, to identify potential ecological associations relevant to conservation and management decisions; therefore, we did not intend to build predictive models or rank predictors based on effect size. Since all predictors were of equal *a priori* interest, formal model comparison was not conducted, consistent with our exploratory objectives. Furthermore, given the risk of inflated Type I errors as all five traps (that represent the responses) at each site shared identical predictor values (original manuscript L288–290, revised L307–309), the relatively small number of sites limited our ability to statistically assess collinearity among predictors and would further increase the risk of biased estimates when more than one predictor was incorporated in the models.

In the Methods, we have clarified our rationale for using bivariate models (L311–313) and noted that we did not rank predictors or compare our individual models (L314). We also present the limitations associated with this approach in the Discussion (L689–695) and emphasize in the Discussion that inferences should be generalized beyond the study locations with care (L675–676).

**Reviewer 3 Comments**

*1. The study makes a relevant and timely contribution, particularly given the limited ecological research on Alnus rubra–dominated systems. By focusing on beetle diversity in red alder ecosystems, the authors address a clear knowledge gap in a habitat increasingly affected by disturbance and fragmentation. The use of beetle bycatch from ethanol-baited traps is both efficient and cost-effective, effectively leveraging existing monitoring efforts targeting ambrosia beetles. This approach aligns well with contemporary trends in integrative and low-impact biodiversity assessment.*

*The study is grounded in a clear, hypothesis-driven framework linking stand conditions to habitat and resource availability. The inclusion of ecologically meaningful variables—such as deadwood, forest continuity, and structural heterogeneity—represents a notable strength, particularly given their well-established importance for saproxylic beetles. Furthermore, the application of appropriate statistical models demonstrates awareness of the distributional properties of count data (e.g., overdispersion), thereby enhancing analytical rigor.*

We thank Reviewer 3 for their supportive feedback. We are proud of this work and are pleased that our hypothesis-driven framework, selection of ecologically meaningful variables, and statistical approach were considered strengths of our study.

Given the scarcity of prior studies on beetle community ecology in red alder-dominated forests in British Columbia, our hypotheses were grounded in general trends from the broader literature on beetle ecology in other forest and terrestrial ecosystems. Our statistical approach was carefully considered to balance our exploratory objectives, the discovery of ecologically associations, risks of inflated Type I error, and specific data distributions of our response variables. We believe that our study provides a solid foundation for future research on beetle ecology and biodiversity studies, particularly saproxylic beetles, in red alder-dominated forests, an ecosystem that harbours a rich invertebrate assemblage yet remains understudied despite its declines from increasing scolytine infestations. We hope this work advocates for the value of low-impact biodiversity assessments in conjunction with existing pest monitoring programs.

*2. However, several methodological and conceptual limitations should be considered when interpreting the results.*

*A primary concern is the limited taxonomic resolution. Identification restricted to the family level substantially constrains ecological inference: it obscures species-specific responses to stand conditions and fragmentation, reduces sensitivity to subtle changes in biodiversity, and may mask contrasting trends within families. This limitation is particularly critical in Coleoptera, where ecological differentiation within families can be considerable. In the same context, the use of Shannon diversity at the family level may inflate perceived diversity patterns and obscure biologically meaningful variation occurring at finer taxonomic scales.*

Please see our response to Reviewer 1 Comment #3 for further details on our rationale for using family-level taxonomic resolution. While species-level resolution would be a valuable complement to our findings, our results demonstrate that family-level resolution can provide meaningful insights into forest conservation and management efforts and is particularly useful for practitioners who are not specialized in beetle taxonomy. Also, rather than producing absolute biodiversity estimates, our study primarily focused on comparisons among structurally distinct study sites to identify which stand attributes or fragmentation level tend to associate with higher beetle diversity relevant to conservation prioritization (L685–689).

Nonetheless, we acknowledge that family-level diversity measures should be interpreted as useful broad indicators of community-level patterns rather than high-resolution biodiversity estimates. We have addressed the limitations of limited taxonomic resolution, including the potential for obscured specific-specific responses (original manuscript L608–616, revised L646–656) and inflated diversity metrics (L683–685), with an additional reference (L685), in the Discussion.

*3. Sampling bias associated with the trap type represents an additional limitation. Ethanol-baited multiple-funnel traps selectively attract specific ecological guilds, particularly xylophagous and saproxylic taxa. As a consequence, the resulting dataset is not representative of the entire beetle community, with underrepresentation of non-attracted groups (e.g., herbivorous or ground-dwelling taxa). Therefore, conclusions regarding beetle diversity should be interpreted as reflecting trap-biased assemblages rather than the full community.*

Please see our response to Reviewer 2 Comment #2 for details on how we addressed the limitation of sampling bias in the Discussion. Briefly, we have added a statement to clarify that our interpretations and conclusions are restricted to flying terrestrial saproxylic beetles (L547–549) and discussed this limitation, including the underrepresentation of certain groups (L676-683).

*4. The relatively small number of study sites (five forests) further limits statistical power and the generalizability of the findings. It also constrains the ability to disentangle potentially correlated environmental variables. In addition, the possibility of pseudoreplication should be explicitly addressed, particularly if multiple traps within sites are not appropriately accounted for in the analytical framework.*

Please see our responses to Academic Editor Comments #1 and 11 for details on how we have addressed the limitation of relatively small number of sites and associated statistical limitations. While we had initially noted non-independence and the inflated risk of Type I errors in the Methods (original manuscript L288–290, revised L307–309), we have now explicitly introduced the limitations of having a small number of study sites and pseudoreplication in the Discussion (L689–698). Since our data and analyses did lead to informative and justifiable conclusions about environmental influences on saproxylic beetle diversity, we do not believe that our sampling design negatively affected the (now restated) conclusions and associated implications of our study.

*5. Temporal limitation is another important issue, as sampling was conducted in a single year (2024). This restricts inference about interannual variability and limits the robustness of conclusions about fragmentation effects. Beetle assemblages are known to exhibit substantial temporal fluctuations driven by climatic and stochastic processes.*

We agree and have introduced this temporal limitation in the Discussion (L699–701), including two additional supporting references (L701).

*6. Although fragmentation is included as a predictor, the use of relatively simple metrics (e.g., connectivity, patch size) may not fully capture landscape complexity. Key processes such as matrix quality, edge effects, and dispersal constraints may therefore be underrepresented, limiting causal interpretation. Relatedly, potential confounding between stand structure and fragmentation should be considered, as these factors are often correlated; without explicit control for collinearity, disentangling their independent effects may be difficult.*

We agree that the interpretation of the effects of fragmentation on red alder beetle communities would benefit from using more complex fragmentation measures; however, this was beyond the scope of our study. Accordingly, we have addressed the limitations associated with the use of relatively simple fragmentation metrics (L656–662), with one additional supporting reference cited on L657.

The issue of collinearity in our study was minimal because we used single-predictor models and interpreted each explanatory variable individually. Regardless, we acknowledge the potential for collinearity between stand conditions and fragmentation and have now introduced this limitation in the Discussion (L692–695).

*7. While the Discussion provides a thorough ecological interpretation of the observed patterns, the study's methodological limitations are only partially acknowledged and not consistently integrated into the interpretation. In particular, the implications of trap selectivity, family-level taxonomic resolution, limited spatial and temporal replication, and simplified habitat characterization remain underdeveloped. As a result, several conclusions—especially those referring broadly to beetle diversity and fragmentation effects—are stronger than warranted by the data. I recommend explicitly consolidating these limitations within the Discussion and aligning the scope of inference more closely with the study design.*

We thank Reviewer 3 for their constructive feedback. Please see our response to the Academic Editor Comment #1 for a detailed overview on how we have integrated the methodological limitations and tempered the conclusions throughout the Discussion. Please also see our response to Reviewer 3 Comments #3, 2, 4, 5, and 6 above on how we addressed the limitations of trap selectivity, family-level taxonomic resolution, limited spatial replication, limited temporal replication, and simplified habitat characterization, respectively. Briefly, we have consolidated the study constraints into a dedicated limitations paragraph (L675–701). Limitations specific to fragmentation were addressed separately within the paragraph on the associations between saproxylic beetle diversity and forest fragmentation, where their placement was deemed to be more appropriate (L646–660).

**Author-initiated Revisions**

The following edits were made to comply with the Creative Commons Attribution License (CC BY 4.0) and improve clarity, without altering the content of the manuscript:

- We have updated the caption for Fig 1 to include appropriate attribution to the USGS National Map Viewer and OpenStreetMap Foundation (L175–179). Also, the inset map is enlarged and the locator map is reduced in size to better highlight the Lower Mainland in Fig 1.
- We have added a photo credit to the captions of Figs 2 and S1 (L205, 1139–1140).
- In Fig 3, we have replaced the Google Earth satellite image with an illustrative diagram that visualizes the formation of the 1 km-radius circular plot used for forest fragmentation measurements, and the figure caption was updated accordingly (L257–263).
- We have removed Fig S2, which contained Google Earth satellite images.

We have also briefly mentioned in the Introduction that the sampling design was primarily used to target ambrosia beetles in red alder forests (L124), for additional context on the source of bycatch. To improve clarity, we have added brief study context to the captions of Figs 2–8 (L200–203, 262–263, 428–431, 446–449, 472–475, 508–511, 530–531), Tables 1–3 (L233–236, 386–389, 486–490), Fig S1 (L1135–1137), and Tables S1–S2 (L1143–1145, 1152–1156). Further, numbered in-text citations and the reference list were updated to reflect changes, consistent with Academic Editor and reviewer recommendations, in the order and the addition of references (please see the revised manuscript with track changes for details).
