# Supplementary material for: Trap tales: The influence of red alder stand conditions and forest fragmentation on family-level beetle bycatch diversity
Source: PLoS One. 2026 Jul 15;21(7):e0353780. doi: 10.1371/journal.pone.0353780 (PMC13372131; doi:10.1371/journal.pone.0353780)
Supplement: Peer review history

# Peer review: Decision Letter 0

**DOI:** 10.1371/journal.pone.0353780.r001

**Authors:** Frank H. Koch

5 Apr 2026

PONE-D-26-07315

Trap tales: The influence of red alder stand conditions and forest fragmentation on family-level beetle bycatch diversity

PLOS One

Dear Dr. Carroll,

Thank you for submitting your manuscript to PLOS ONE. After careful consideration, we feel that it has merit but does not fully meet PLOS ONE’s publication criteria as it currently stands. Therefore, we invite you to submit a revised version of the manuscript that addresses the points raised during the review process.

I've received comments from three reviewers. Based on their assessments as well as my own, I believe that your manuscript may be suitable for publication with a moderate level of revision. I agree with you (as do all the reviewers) about the information value of insect trap bycatch, and so I applaud you for undertaking this effort. I think the main criticism that emerges from the review comments is that you need to provide a fuller account of the methodological limitations of the study in your Discussion. You should also reexamine your conclusions, some of which should probably be tempered due to these limitations. Additionally, some aspects of your manuscript would benefit from clarification -- please look to the specific comments for guidance in that regard. Again, the reviewers and I see the merits of your manuscript, and because it is well-written and organized overall, I would be happy to reconsider it if you make the necessary changes.

Please submit your revised manuscript by May 20 2026 11:59PM. If you will need more time than this to complete your revisions, please reply to this message or contact the journal office at plosone@plos.org. When you're ready to submit your revision, log on to https://www.editorialmanager.com/pone/ and select the 'Submissions Needing Revision' folder to locate your manuscript file.

Please include the following items when submitting your revised manuscript:

- A letter that responds to each point raised by the academic editor and reviewer(s). You should upload this letter as a separate file labeled 'Response to Reviewers'.
- A marked-up copy of your manuscript that highlights changes made to the original version. You should upload this as a separate file labeled 'Revised Manuscript with Track Changes'.
- An unmarked version of your revised paper without tracked changes. You should upload this as a separate file labeled 'Manuscript'.

If you would like to make changes to your financial disclosure, please include your updated statement in your cover letter. Guidelines for resubmitting your figure files are available below the reviewer comments at the end of this letter.

If applicable, we recommend that you deposit your laboratory protocols in protocols.io to enhance the reproducibility of your results. Protocols.io assigns your protocol its own identifier (DOI) so that it can be cited independently in the future. For instructions see: https://journals.plos.org/plosone/s/submission-guidelines#loc-laboratory-protocols. Additionally, PLOS ONE offers an option for publishing peer-reviewed Lab Protocol articles, which describe protocols hosted on protocols.io. Read more information on sharing protocols at https://plos.org/protocols?utm_medium=editorial-email&utm_source=authorletters&utm_campaign=protocols.

As the corresponding author, your ORCID iD is verified in the submission system and will appear in the published article. PLOS supports the use of ORCID, and we encourage all coauthors to register for an ORCID iD and use it as well. Please encourage your coauthors to verify their ORCID iD within the submission system before final acceptance, as unverified ORCID iDs will not appear in the published article. *Only*  the individual author can complete the verification step; PLOS staff *cannot*  verify ORCID iDs on behalf of authors.

We look forward to receiving your revised manuscript.

Kind regards,

Frank H. Koch, PhD

Academic Editor

PLOS One

**Journal Requirements:**

When submitting your revision, we need you to address these additional requirements.

1. Please ensure that your manuscript meets PLOS ONE's style requirements, including those for file naming. The PLOS ONE style templates can be found at

https://journals.plos.org/plosone/s/file?id=wjVg/PLOSOne_formatting_sample_main_body.pdf and

https://journals.plos.org/plosone/s/file?id=ba62/PLOSOne_formatting_sample_title_authors_affiliations.pdf

2. In the online submission form, you indicated that your data will be submitted to a repository upon acceptance.  We strongly recommend all authors deposit their data before acceptance, as the process can be lengthy and hold up publication timelines. Please note that, though access restrictions are acceptable now, your entire minimal  dataset will need to be made freely accessible if your manuscript is accepted for publication. This policy applies to all data except where public deposition would breach compliance with the protocol approved by your research ethics board. If you are unable to adhere to our open data policy, please kindly revise your statement to explain your reasoning and we will seek the editor's input on an exemption.

3. We note that Figures 1, 3 and Figure S2 in your submission contain map and satellite images which may be copyrighted. All PLOS content is published under the Creative Commons Attribution License (CC BY 4.0), which means that the manuscript, images, and Supporting Information files will be freely available online, and any third party is permitted to access, download, copy, distribute, and use these materials in any way, even commercially, with proper attribution. For these reasons, we cannot publish previously copyrighted maps or satellite images created using proprietary data, such as Google software (Google Maps, Street View, and Earth). For more information, see our copyright guidelines: http://journals.plos.org/plosone/s/licenses-and-copyright.

We require you to either (1) present written permission from the copyright holder to publish these figures specifically under the CC BY 4.0 license, or (2) remove the figures from your submission:

1. You may seek permission from the original copyright holder of Figures 1, 3 and Figure S2 to publish the content specifically under the CC BY 4.0 license.

We recommend that you contact the original copyright holder with the Content Permission Form (http://journals.plos.org/plosone/s/file?id=7c09/content-permission-form.pdf) and the following text:

“I request permission for the open-access journal PLOS ONE to publish XXX under the Creative Commons Attribution License (CCAL) CC BY 4.0 (http://creativecommons.org/licenses/by/4.0/). Please be aware that this license allows unrestricted use and distribution, even commercially, by third parties. Please reply and provide explicit written permission to publish XXX under a CC BY license and complete the attached form.”

Please upload the completed Content Permission Form or other proof of granted permissions as an "Other" file with your submission.

In the figure caption of the copyrighted figure, please include the following text: “Reprinted from [ref] under a CC BY license, with permission from [name of publisher], original copyright [original copyright year].”

2. If you are unable to obtain permission from the original copyright holder to publish these figures under the CC BY 4.0 license or if the copyright holder’s requirements are incompatible with the CC BY 4.0 license, please either i) remove the figure or ii) supply a replacement figure that complies with the CC BY 4.0 license. Please check copyright information on all replacement figures and update the figure caption with source information. If applicable, please specify in the figure caption text when a figure is similar but not identical to the original image and is therefore for illustrative purposes only.

The following resources for replacing copyrighted map figures may be helpful:

USGS National Map Viewer (public domain): http://viewer.nationalmap.gov/viewer/

The Gateway to Astronaut Photography of Earth (public domain): http://eol.jsc.nasa.gov/sseop/clickmap/

Maps at the CIA (public domain): https://www.cia.gov/library/publications/the-world-factbook/index.html and https://www.cia.gov/library/publications/cia-maps-publications/index.html

NASA Earth Observatory (public domain): http://earthobservatory.nasa.gov/

Landsat: http://landsat.visibleearth.nasa.gov/

USGS EROS (Earth Resources Observatory and Science (EROS) Center) (public domain): http://eros.usgs.gov/#

Natural Earth (public domain): http://www.naturalearthdata.com/

4.  We note that Figure 2, and Figure S1 in your submission contain copyrighted images. All PLOS content is published under the Creative Commons Attribution License (CC BY 4.0), which means that the manuscript, images, and Supporting Information files will be freely available online, and any third party is permitted to access, download, copy, distribute, and use these materials in any way, even commercially, with proper attribution. For more information, see our copyright guidelines: http://journals.plos.org/plosone/s/licenses-and-copyright.

We require you to either (1) present written permission from the copyright holder to publish these figures specifically under the CC BY 4.0 license, or (2) remove the figures from your submission:

1. You may seek permission from the original copyright holder of Figure 2 and Figure S1 to publish the content specifically under the CC BY 4.0 license.

We recommend that you contact the original copyright holder with the Content Permission Form (http://journals.plos.org/plosone/s/file?id=7c09/content-permission-form.pdf) and the following text:

“I request permission for the open-access journal PLOS ONE to publish XXX under the Creative Commons Attribution License (CCAL) CC BY 4.0 (http://creativecommons.org/licenses/by/4.0/). Please be aware that this license allows unrestricted use and distribution, even commercially, by third parties. Please reply and provide explicit written permission to publish XXX under a CC BY license and complete the attached form.”

Please upload the completed Content Permission Form or other proof of granted permissions as an "Other" file with your submission.

In the figure caption of the copyrighted figure, please include the following text: “Reprinted from [ref] under a CC BY license, with permission from [name of publisher], original copyright [original copyright year].”

2. If you are unable to obtain permission from the original copyright holder to publish these figures under the CC BY 4.0 license or if the copyright holder’s requirements are incompatible with the CC BY 4.0 license, please either i) remove the figure or ii) supply a replacement figure that complies with the CC BY 4.0 license. Please check copyright information on all replacement figures and update the figure caption with source information. If applicable, please specify in the figure caption text when a figure is similar but not identical to the original image and is therefore for illustrative purposes only.

5. Please upload a new copy of Figures 1, 4, 5, 6, 7 and 8, as the detail is not clear. Please follow the link for more information:  https://journals.plos.org/plosone/s/figures

6. If the reviewer comments include a recommendation to cite specific previously published works, please review and evaluate these publications to determine whether they are relevant and should be cited. There is no requirement to cite these works unless the editor has indicated otherwise.

**Additional Editor Comments:**

L17: insert comma after "diversity"

L81: should be "promotes" instead of "promote"

L202-203 ("...and dead if foliage was absent for deciduous trees ..."): This implies that the stands were inventoried in leaf-on conditions. Was that the case? I would indicate in the text.

L208-211: So, in retrospect, would a different plot configuration have been better? For example, three smaller fixed-area plots at each location. Or would the result have been essentially the same? This is primarily a point for discussion (i.e., plot configuration matters, perhaps especially with small sample sizes).

L289-292: I agree that it makes sense to prioritize minimizing false negatives.

L345: use "spp." instead of "sp."

L349: replace "comprising" with "comprised of"

L376-378: Did the five sites differ noticeably in the main drivers of fragmentation? For instance, MK was the most remote but had a moderate level of forestry-related fragmentation. Were any of other sites similar? (Another point for discussion.)

L385-387: See previous comment. Did BL have any forestry activity?

L388-392: I do worry somewhat that five sites are not sufficiently representative of the landscape diversity. (Again, a point for discussion.)

L459-465: I'm not sure we can put too much stock in these results. 60% of the traps were in sites where there were no dead red alders recorded in the forest plot, so they're all represented as zeros even if there was some variation in dead red alder presence within the larger area. Again, in retrospect, would you have been better served by a different inventory plot configuration?

L478-479: I think we can put more stock in these results, given the between-site variation.

[Note: HTML markup is below. Please do not edit.]

Reviewers' comments:

Reviewer's Responses to Questions

**Comments to the Author**

1. Is the manuscript technically sound, and do the data support the conclusions?

The manuscript must describe a technically sound piece of scientific research with data that supports the conclusions. Experiments must have been conducted rigorously, with appropriate controls, replication, and sample sizes. The conclusions must be drawn appropriately based on the data presented.

Reviewer #1: Yes

Reviewer #2: Yes

Reviewer #3: Partly

**********

2. Has the statistical analysis been performed appropriately and rigorously?

Reviewer #1: Yes

Reviewer #2: Yes

Reviewer #3: Yes

**********

3. Have the authors made all data underlying the findings in their manuscript fully available?

The PLOS Data policy requires authors to make all data underlying the findings described in their manuscript fully available without restriction, with rare exception (please refer to the Data Availability Statement in the manuscript PDF file). The data should be provided as part of the manuscript or its supporting information, or deposited to a public repository. For example, in addition to summary statistics, the data points behind means, medians and variance measures should be available. If there are restrictions on publicly sharing data—e.g. participant privacy or use of data from a third party—those must be specified.

Reviewer #1: Yes

Reviewer #2: Yes

Reviewer #3: Yes

**********

4. Is the manuscript presented in an intelligible fashion and written in standard English?

PLOS ONE does not copyedit accepted manuscripts, so the language in submitted articles must be clear, correct, and unambiguous. Any typographical or grammatical errors should be corrected at revision, so please note any specific errors here.

Reviewer #1: Yes

Reviewer #2: Yes

Reviewer #3: Yes

**********

5. Review Comments to the Author

Please use the space provided to explain your answers to the questions above. You may also include additional comments for the author, including concerns about dual publication, research ethics, or publication ethics. (Please upload your review as an attachment if it exceeds 20,000 characters)

Reviewer #1: I have a few separate comments for the authors:

- Redo the abstract. Specify exact numbers, not abstract words.

- The introduction lacks information about beetle baits based on beer, sugar, ethanol, and other agents. See such publications (https://doi.org/10.1134/S0013873824010056; https://doi.org/10.1007/s10841-017-9965-3; https://doi.org/10.1649/0010-065X-74.4.763; https://doi.org/10.3390/insects15060420) and give their analysis separately.

- Why weren't the beetles identified before the species?

- Why do I need Figure 2? It shows nothing and is of very poor quality.

- Make a conclusion

Reviewer #2: I enjoyed reading the manuscript about beetle family diversity in red alder ecosystems. It is well written and structured. Methods and analyses seem very solid.

I appreciate the authors’ use of bycatch from existing insect traps for ecological analysis. I agree that bycatch deserves ethical consideration and can be a valuable and cost effective source of data for many ecological research questions.

I also recognize the limitations of this sampling method. Because these traps are designed mainly to catch flying, terrestrial insects, some beetle families are likely underrepresented or missed entirely. For example, mostly ground dwelling or flightless groups, such as Carabidae, are unlikely to be sampled well using this approach.

I therefore suggest adding a clearly defined paragraph or section that explicitly discusses the limitations of the study and of this sampling method. Although these limitations are mentioned briefly, especially in the first paragraph of the Discussion, I think they should be stated more clearly and in a more structured way. This would improve transparency and help readers better understand how broadly the results can be interpreted.

Regarding the first paragraph of the Discussion, I suggest revising it substantially. Much of the information presented there seems more appropriate for the Results section. For example, the Discussion repeats which beetle families were captured as bycatch, even though this information is already presented in the Results and Table S2. The Results section already reports the total number of beetles and families captured, as well as the most abundant families. The Discussion would be stronger if this repetition were reduced and the focus shifted toward interpretation, including methodological limitations such as possible taxonomic biases in sampling.

I also have a question about the field measurements. Were all stand level metrics collected by the same researcher? If not, how was observer bias addressed? This is especially relevant for measurements such as DBH, where small differences among observers could affect the results. Clarifying this would increase confidence in the reliability of the field data.

The authors use single predictor models to assess the relationship between stand conditions, fragmentation metrics and beetle diversity. While this approach is useful for exploratory analyses, it does not allow assessment of the relative importance of predictors or their effects after accounting for correlations among variables.

Could the authors clarify why multivariate models were not used, for example after checking collinearity among predictors? Including multiple predictors in a single model (with standardized coefficients) would allow direct comparison of effect sizes and help identify which variables have the strongest associations with richness or diversity.

If the intention is not to rank predictors, this limitation should be stated more explicitly. It would also be helpful to clarify whether single predictor models were compared (e.g. using AICc), and what conclusions can reasonably be drawn from such comparisons.

Reviewer #3: The study makes a relevant and timely contribution, particularly given the limited ecological research on Alnus rubra–dominated systems. By focusing on beetle diversity in red alder ecosystems, the authors address a clear knowledge gap in a habitat increasingly affected by disturbance and fragmentation. The use of beetle bycatch from ethanol-baited traps is both efficient and cost-effective, effectively leveraging existing monitoring efforts targeting ambrosia beetles. This approach aligns well with contemporary trends in integrative and low-impact biodiversity assessment.

The study is grounded in a clear, hypothesis-driven framework linking stand conditions to habitat and resource availability. The inclusion of ecologically meaningful variables—such as deadwood, forest continuity, and structural heterogeneity—represents a notable strength, particularly given their well-established importance for saproxylic beetles. Furthermore, the application of appropriate statistical models demonstrates awareness of the distributional properties of count data (e.g., overdispersion), thereby enhancing analytical rigor.

However, several methodological and conceptual limitations should be considered when interpreting the results.

A primary concern is the limited taxonomic resolution. Identification restricted to the family level substantially constrains ecological inference: it obscures species-specific responses to stand conditions and fragmentation, reduces sensitivity to subtle changes in biodiversity, and may mask contrasting trends within families. This limitation is particularly critical in Coleoptera, where ecological differentiation within families can be considerable. In the same context, the use of Shannon diversity at the family level may inflate perceived diversity patterns and obscure biologically meaningful variation occurring at finer taxonomic scales.

Sampling bias associated with the trap type represents an additional limitation. Ethanol-baited multiple-funnel traps selectively attract specific ecological guilds, particularly xylophagous and saproxylic taxa. As a consequence, the resulting dataset is not representative of the entire beetle community, with underrepresentation of non-attracted groups (e.g., herbivorous or ground-dwelling taxa). Therefore, conclusions regarding beetle diversity should be interpreted as reflecting trap-biased assemblages rather than the full community.

The relatively small number of study sites (five forests) further limits statistical power and the generalizability of the findings. It also constrains the ability to disentangle potentially correlated environmental variables. In addition, the possibility of pseudoreplication should be explicitly addressed, particularly if multiple traps within sites are not appropriately accounted for in the analytical framework.

Temporal limitation is another important issue, as sampling was conducted in a single year (2024). This restricts inference about interannual variability and limits the robustness of conclusions about fragmentation effects. Beetle assemblages are known to exhibit substantial temporal fluctuations driven by climatic and stochastic processes.

Although fragmentation is included as a predictor, the use of relatively simple metrics (e.g., connectivity, patch size) may not fully capture landscape complexity. Key processes such as matrix quality, edge effects, and dispersal constraints may therefore be underrepresented, limiting causal interpretation. Relatedly, potential confounding between stand structure and fragmentation should be considered, as these factors are often correlated; without explicit control for collinearity, disentangling their independent effects may be difficult.

While the Discussion provides a thorough ecological interpretation of the observed patterns, the study's methodological limitations are only partially acknowledged and not consistently integrated into the interpretation. In particular, the implications of trap selectivity, family-level taxonomic resolution, limited spatial and temporal replication, and simplified habitat characterization remain underdeveloped. As a result, several conclusions—especially those referring broadly to beetle diversity and fragmentation effects—are stronger than warranted by the data. I recommend explicitly consolidating these limitations within the Discussion and aligning the scope of inference more closely with the study design.

**********

6. PLOS authors have the option to publish the peer review history of their article (what does this mean?). If published, this will include your full peer review and any attached files.

If you choose “no”, your identity will remain anonymous but your review may still be made public.

**Do you want your identity to be public for this peer review?**  For information about this choice, including consent withdrawal, please see our Privacy Policy.

Reviewer #1: No

Reviewer #2: No

Reviewer #3: No

**********

[NOTE: If reviewer comments were submitted as an attachment file, they will be attached to this email and accessible via the submission site. Please log into your account, locate the manuscript record, and check for the action link "View Attachments". If this link does not appear, there are no attachment files.]

To ensure your figures meet our technical requirements, please review our figure guidelines: https://journals.plos.org/plosone/s/figures

You may also use PLOS’s free figure tool, NAAS, to help you prepare publication quality figures: https://journals.plos.org/plosone/s/figures#loc-tools-for-figure-preparation.

NAAS will assess whether your figures meet our technical requirements by comparing each figure against our figure specifications.

# Author comment: Author response to Decision Letter 1

**DOI:** 10.1371/journal.pone.0353780.r002

29 May 2026

25 May 2026

Dear Dr. Frank Koch, Academic Editor, PLOS One,

We, Ting Ting Michelle Yim, Sichen Zhou, Debra Wertman, and Allan Carroll, the authors of “Trap tales: The influence of red alder stand conditions and forest fragmentation on family-level beetle bycatch diversity” (PONE-D-26-07315), thank the editor and reviewers for their constructive feedback on our manuscript. This letter addresses the Academic Editor’s and reviewers’ comments, indicating where and how the suggestions were incorporated to greatly improve our manuscript.

Editor and Reviewer comments are listed with our responses following each. Unless otherwise stated, line numbers referenced by the reviewers correspond to the submitted manuscript, while the line numbers we referenced correspond to the revised manuscript (clean version).

Academic Editor Comments

1. I agree with you (as do all the reviewers) about the information value of insect trap bycatch, and so I applaud you for undertaking this effort. I think the main criticism that emerges from the review comments is that you need to provide a fuller account of the methodological limitations of the study in your Discussion. You should also reexamine your conclusions, some of which should probably be tempered due to these limitations. Additionally, some aspects of your manuscript would benefit from clarification -- please look to the specific comments for guidance in that regard. Again, the reviewers and I see the merits of your manuscript, and because it is well-written and organized overall, I would be happy to reconsider it if you make the necessary changes.

We thank the editor and reviewers for recognizing the significance of bycatch in ecological research and for their constructive feedback. Our response here is a summary of the major comments we have addressed.

Overall, we have added a clearer, more tempered account of our methodological limitations in the Discussion, including a dedicated limitations paragraph. We present the following summary of the specific methodological limitations raised by the Academic Editor and reviewers (please see our responses to the specific comments below for additional details):

(1) Sampling bias towards saproxylic taxa: While we had initially commented on this issue in the Discussion (original manuscript L511–517, revised L676–683), we have now added a cautionary statement on data interpretation related to this inherent sampling bias (L547–549).

(2) Coarse taxonomic resolution: We retain our initial perspective that family-level diversity metrics are useful and informative for analyses of ecosystem structure and function (original manuscript L645–648, revised L711–714), but have now expanded on our choice of family-level resolution for this study in the Introduction (original manuscript L54–58, revised L55–65) and further discuss our rationale in the Discussion (L714–719).

While we had initially indicated that the family-level resolution implemented in our study may hinder underlying species turnover processes in relation to fragmentation (original manuscript L608–616, revised L646–654), we have now added text clarifying that family-level resolution may inflate perceived diversity indices (L683–685) and that finer resolutions would be suitable for inferring within-family compositional shifts (L654–656).

(3) Small sample size (traps and plots within sites) and related statistical constraints: In the revised Discussion, we have explicitly mentioned that the relatively small number of sampling sites (5) and traps (5 per site), and the fixed-area plot configuration, may have incompletely captured landscape diversity and constrained our statistical analyses, including preventing the use of multivariable models (L689–695).

We had initially stated that our measurement of dead red alder availability represents a conservative estimate due to the limited number of sites (2) where dead red alders were observed in fixed-area plots (original manuscript L208–211, revised L225–228), and noted non-independence and inflated risk of Type I errors in the Methods (original manuscript L288–290, revised L307–309). We have now expanded on these limitations in the Discussion (L689–698). We have clarified the reasons for adopting bivariate models (i.e., models with one predictor and one response variable) in the Methods (L311–313) and Discussion (L692–695). We also now specify in the Methods that no predictor ranking or model comparison were conducted (L314) and indicate in the Discussion that results should therefore be interpreted as exploratory (L697–698).

(4) Temporal limitation: We have explicitly stated that our study represents a temporal snapshot and does not indicate interannual variability (L699–701).

(5) Fragmentation metrics: We have clarified that the use of relatively simple fragmentation metrics limits causal interpretations (L656–660).

2. L17: insert comma after "diversity"

We have made this edit (L17).

3. L81: should be "promotes" instead of "promote"

We have made this edit (L88).

4. L202-203 ("...and dead if foliage was absent for deciduous trees ..."): This implies that the stands were inventoried in leaf-on conditions. Was that the case? I would indicate in the text.

All stands were inventoried in leaf-on conditions in early to mid-August 2024. We have indicated this in the revised manuscript (L209–210).

5. L208-211: So, in retrospect, would a different plot configuration have been better? For example, three smaller fixed-area plots at each location. Or would the result have been essentially the same? This is primarily a point for discussion (i.e., plot configuration matters, perhaps especially with small sample sizes).

We thank the Academic Editor for this thoughtful comment. The use of the 11.28 m-radius fixed-area plot configuration is a conventional approach used by the Canadian National Forest Inventory Program (cited on original manuscript L193, revised L209) and provincial forest resource monitoring programs in British Columbia (e.g., Provincial Change Monitoring Inventory, ground-truthing for Vegetation Resources Inventory). It is applied to estimate forest stand conditions of forests dominated by large trees defined by diameter at breast height (DBH) ≥ 9 cm at a broad geographical scale. In our study, we applied a slightly lower threshold (DBH ≥ 8 cm) intended to accommodate smaller red alder trees, following Wertman et al. (2025), which we have now cited on L214.

However, we acknowledge that the plot configuration and a single plot per site could not fully capture the variability present within declining red alder stands, and thus the presence of standing dead red alders were missed at three sites. We have revised the Discussion to address this limitation (L551–553, 689–692).

6. L289-292: I agree that it makes sense to prioritize minimizing false negatives.

We thank the editor for acknowledging our analytical approach.

7. L345: use "spp." instead of "sp."

We have made this edit (L373).

8. L349: replace "comprising" with "comprised of"

We have made this edit (L377).

9. L376-378: Did the five sites differ noticeably in the main drivers of fragmentation? For instance, MK was the most remote but had a moderate level of forestry-related fragmentation. Were any of other sites similar? (Another point for discussion.)

Yes, the five sites differed with respect to main drivers of forest fragmentation. All sites, except MK, do not have forestry activities as they are managed for conservation, education, and recreation under the Metro Vancouver Regional Parks Plan (https://metrovancouver.org/services/regional-parks/regional-parks-plan), rather than for timber harvest and other forestry activities. We have noted that fragmentation drivers were identified from Google Earth satellite imagery in the Methods (L247–248), clarified the site-specific fragmentation drivers in the Results (L402–405, 407–409, 414), and mentioned them briefly in relation to beetle diversity and abundances in the Discussion (L620–628, 630–631).

10. L385-387: See previous comment. Did BL have any forestry activity?

BL does not have any forestry activity (L414), although it has a comparable number of forest patches and mean edge-to-area ratio as MK where fragmentation is primarily driven by forestry. We have now clarified the fragmentation drivers and indicate reasons for its lowest total forest cover in the Results (L404–405, 407–409).

11. L388-392: I do worry somewhat that five sites are not sufficiently representative of the landscape diversity. (Again, a point for discussion.)

Although our sample size was limited to five sites, these locations were carefully selected to capture a range of forest landscapes spanning naturalized to suburban/urban conditions in the Lower Mainland of British Columbia. However, we acknowledge that our sample size may not completely represent the full spectrum of the landscape diversity of red alder forests that beetles inhabit and may limit the generalizability of our conclusions. We have revised the Discussion to address this limitation (L689–692).

12. L459-465: I'm not sure we can put too much stock in these results. 60% of the traps were in sites where there were no dead red alders recorded in the forest plot, so they're all represented as zeros even if there was some variation in dead red alder presence within the larger area. Again, in retrospect, would you have been better served by a different inventory plot configuration?

Please see our response to Academic Editor Comment #5 for the rationale for the plot configuration used and how this limitation was incorporated in the Discussion. We have tempered the language of our conclusions regarding dead red alder availability in the Discussion (L551–552, 556, 558, 560, 565, 570–571, 575).

13. L478-479: I think we can put more stock in these results, given the between-site variation.

We agree with the Academic Editor on this point and have expanded upon our conclusions in the Discussion regarding between-site variation in fragmentation drivers in relation to diversity and abundances (L620–631).

Reviewer 1 Comments

1. Redo the abstract. Specify exact numbers, not abstract words.

We have revised the Abstract to include additional quantitative information (L25–28), more precise conclusions (L33–36), and a cautionary note regarding the limitations of the study (L38–39).

2. The introduction lacks information about beetle baits based on beer, sugar, ethanol, and other agents. See such publications (https://doi.org/10.1134/S0013873824010056; https://doi.org/10.1007/s10841-017-9965-3; https://doi.org/10.1649/0010-065X-74.4.763; https://doi.org/10.3390/insects15060420) and give their analysis separately.

We thank Reviewer 1 for this suggestion. The use of semiochemical baits in forest beetle pest monitoring is already described in the Introduction (original manuscript L100–102, revised L107–19). We have further introduced ethanol (L110–112), a kairomone widely used in forest scolytine monitoring (including in this study), as well as beer, wine, and sugar (L112–115), citing two of the recommended references (L114), in the revised Introduction.

3. Why weren't the beetles identified before the species?

We have interpreted this comment as “Why weren’t the beetles identified to species?” We implemented family-level identification as a practical and efficient method to assess broad beetle community patterns to inform management strategies, which is especially useful when expertise, time, and financial limitations are present in ecological studies. Our results showed that family-level resolution was indeed informative for detecting important ecological associations of the saproxylic beetle community in red alder forests and hence valuable for conservation and management applications.

We had initially outlined the rationale for using family-level identification in the original manuscript (L54–58) but have now expanded on and clarified this rationale in the revised Introduction (L55–65) and Discussion (L711–719), respectively. We have also explicitly addressed the limitations of this approach in the Discussion (L683–689). We have added additional supporting references to the Introduction and Discussion to support these revisions (L56–59, 64–65, 685, 719).

4. Why do I need Figure 2? It shows nothing and is of very poor quality.

Figure 2 presents five beetle families, each containing scolytine predators, that were evaluated for their responses to stand conditions and forest fragmentation. This figure is intended to provide a visual reference to support interpretation of the results for readers who work in forest, biodiversity, and conservation management who may not be taxonomic specialists in these beetle families. For this reason, we have retained Figure 2 in the revised manuscript and ensured that it meets the journal requirements.

5. Make a conclusion

The final paragraph of the Discussion presents our conclusions (L702–728). It summarizes our findings and demonstrates the practical implications of family-level identification and the ecological significance of bycatch. If the journal prefers, we can move this paragraph to a separate Conclusion section, but we do like how this information currently flows from the main content of the Discussion.

Reviewer 2 Comments

1. I enjoyed reading the manuscript about beetle family diversity in red alder ecosystems. It is well written and structured. Methods and analyses seem very solid.

I appreciate the authors’ use of bycatch from existing insect traps for ecological analysis. I agree that bycatch deserves ethical consideration and can be a valuable and cost effective source of data for many ecological research questions.

We thank Reviewer 2 for their positive feedback on our manuscript.

2. I also recognize the limitations of this sampling method. Because these traps are designed mainly to catch flying, terrestrial insects, some beetle families are likely underrepresented or missed entirely. For example, mostly ground dwelling or flightless groups, such as Carabidae, are unlikely to be sampled well using this approach.

I therefore suggest adding a clearly defined paragraph or section that explicitly discusses the limitations of the study and of this sampling method. Although these limitations are mentioned briefly, especially in the first paragraph of the Discussion, I think they should be stated more clearly and in a more structured way. This would improve transparency and help readers better understand how broadly the results can be interpreted.

We thank Reviewer 2 for this suggestion. While we had initially commented on the issue of sampling bias in the Discussion (original manuscript L511–517, revised L676–683), we have now consolidated this into a dedicated limitations paragraph (L675–701) and added a cautionary statement noting that data interpretation is limited to flying terrestrial saproxylic beetles (L547–549). Please see our response to the Academic Editor Comment #1 for details on how each limitation was addressed in the revised manuscript.

3. Regarding the first paragraph of the Discussion, I suggest revising it substantially. Much of the information presented there seems more appropriate for the Results section. For example, the Discussion repeats which beetle families were captured as bycatch, even though this information is already presented in the Results and Table S2. The Results section already reports the total number of beetles and families captured, as well as the most abundant families. The Discussion would be stronger if this repetition were reduced and the focus shifted toward interpretation, including methodological limitations such as possible taxonomic biases in sampling.

We thank Reviewer 2 for this observation. We have moved the information on captured beetle families from the first paragraph of the Discussion (original manuscript L501–506, 508–511) to the Results (L353–357, 360–364). We have removed Laemophloeidae and Silvanidae as examples of predatory families (original manuscript L509) as their predatory status is described as potential rather than confirmed in Kenis et al. (2007) (cited on original manuscript L509, revised

Attachment Submitted filename: Yim et al_2026_Response to Reviewers.docx

# Peer review: Decision Letter 1

**DOI:** 10.1371/journal.pone.0353780.r003

**Authors:** Frank H. Koch

29 Jun 2026

Trap tales: The influence of red alder stand conditions and forest fragmentation on family-level beetle bycatch diversity

PONE-D-26-07315R1

Dear Dr. Carroll,

We’re pleased to inform you that your manuscript has been judged scientifically suitable for publication and will be formally accepted for publication once it meets all outstanding technical requirements.

Within one week, you’ll receive an e-mail detailing the required amendments. When these have been addressed, you’ll receive a formal acceptance letter and your manuscript will be scheduled for publication.

An invoice will be generated when your article is formally accepted. Please note, if your institution has a publishing partnership with PLOS and your article meets the relevant criteria, all or part of your publication costs will be covered. Please make sure your user information is up-to-date by logging into Editorial Manager at Editorial Manager® and clicking the ‘Update My Information' link at the top of the page. For questions related to billing, please contact billing support.

If your institution or institutions have a press office, please notify them about your upcoming paper to help maximize its impact. If they’ll be preparing press materials, please inform our press team as soon as possible -- no later than 48 hours after receiving the formal acceptance. Your manuscript will remain under strict press embargo until 2 pm Eastern Time on the date of publication. For more information, please contact onepress@plos.org.

Kind regards,

Frank H. Koch, PhD

Academic Editor

PLOS One

Additional Editor Comments (optional):

I've received comments from two reviewers, both of whom also evaluated your initial submission. The three of us agree that your revised manuscript is an improvement over the initial version. You clearly took some care in responding to the reviewer comments. In particular, I appreciate the additional text in the Discussion regarding your study's limitations, which in my view do not really diminish your findings nor your larger message about the value of bycatch.

Reviewers' comments:

Reviewer's Responses to Questions

**Comments to the Author**

1. If the authors have adequately addressed your comments raised in a previous round of review and you feel that this manuscript is now acceptable for publication, you may indicate that here to bypass the “Comments to the Author” section, enter your conflict of interest statement in the “Confidential to Editor” section, and submit your "Accept" recommendation.

Reviewer #2: All comments have been addressed

Reviewer #3: (No Response)

**********

2. Is the manuscript technically sound, and do the data support the conclusions?

The manuscript must describe a technically sound piece of scientific research with data that supports the conclusions. Experiments must have been conducted rigorously, with appropriate controls, replication, and sample sizes. The conclusions must be drawn appropriately based on the data presented.

Reviewer #2: Yes

Reviewer #3: Yes

**********

3. Has the statistical analysis been performed appropriately and rigorously?

Reviewer #2: Yes

Reviewer #3: Yes

**********

4. Have the authors made all data underlying the findings in their manuscript fully available?

The PLOS Data policy requires authors to make all data underlying the findings described in their manuscript fully available without restriction, with rare exception (please refer to the Data Availability Statement in the manuscript PDF file). The data should be provided as part of the manuscript or its supporting information, or deposited to a public repository. For example, in addition to summary statistics, the data points behind means, medians and variance measures should be available. If there are restrictions on publicly sharing data—e.g. participant privacy or use of data from a third party—those must be specified.

Reviewer #2: Yes

Reviewer #3: (No Response)

**********

5. Is the manuscript presented in an intelligible fashion and written in standard English?

PLOS ONE does not copyedit accepted manuscripts, so the language in submitted articles must be clear, correct, and unambiguous. Any typographical or grammatical errors should be corrected at revision, so please note any specific errors here.

Reviewer #2: Yes

Reviewer #3: Yes

**********

6. Review Comments to the Author

Please use the space provided to explain your answers to the questions above. You may also include additional comments for the author, including concerns about dual publication, research ethics, or publication ethics. (Please upload your review as an attachment if it exceeds 20,000 characters)

Reviewer #2: (No Response)

Reviewer #3: The authors appear to have addressed all of the major concerns raised in my review. They have added clearer acknowledgments of the limitations associated with taxonomic resolution, trap selectivity, limited spatial and temporal replication, simplified fragmentation metrics, and potential pseudoreplication. They have also appropriately narrowed the interpretation of their findings to flying terrestrial saproxylic beetles and clarified that the study provides comparative insights rather than a complete assessment of beetle biodiversity. The revisions improve the transparency and balance of the manuscript, and the authors have adequately incorporated the requested methodological caveats into the Discussion.

I have no further major concerns. The manuscript has been substantially improved and is suitable for publication after these revisions. I recommend acceptance.

I also hope that, in the future, the authors will be able to process this valuable material further and achieve species-level identification, enabling a more detailed assessment of community composition and species-specific ecological responses. I wish them success in this important continuation of their work.

**********

7. PLOS authors have the option to publish the peer review history of their article (what does this mean?). If published, this will include your full peer review and any attached files.

If you choose “no”, your identity will remain anonymous but your review may still be made public.

**Do you want your identity to be public for this peer review?**  For information about this choice, including consent withdrawal, please see our Privacy Policy.

Reviewer #2: No

Reviewer #3: No

**********

# Editor report: Acceptance letter

**DOI:** 10.1371/journal.pone.0353780.r004

**Authors:** Frank H. Koch

PONE-D-26-07315R1

PLOS One

Dear Dr. Carroll,

I'm pleased to inform you that your manuscript has been deemed suitable for publication in PLOS One. Congratulations! Your manuscript is now being handed over to our production team.

At this stage, our production department will prepare your paper for publication. This includes ensuring the following:

* All references, tables, and figures are properly cited

* All relevant supporting information is included in the manuscript submission,

* There are no issues that prevent the paper from being properly typeset

You will receive further instructions from the production team, including instructions on how to review your proof when it is ready. Please keep in mind that we are working through a large volume of accepted articles, so please give us a few days to review your paper and let you know the next and final steps.

Lastly, if your institution or institutions have a press office, please let them know about your upcoming paper now to help maximize its impact. If they'll be preparing press materials, please inform our press team within the next 48 hours. Your manuscript will remain under strict press embargo until 2 pm Eastern Time on the date of publication. For more information, please contact onepress@plos.org.

You will receive an invoice from PLOS for your publication fee after your manuscript has reached the completed accept phase. If you receive an email requesting payment before acceptance or for any other service, this may be a phishing scheme. Learn how to identify phishing emails and protect your accounts at https://explore.plos.org/phishing.

If we can help with anything else, please email us at customercare@plos.org.

Thank you for submitting your work to PLOS ONE and supporting open access.

Kind regards,

PLOS ONE Editorial Office Staff

on behalf of

Dr. Frank H. Koch

Academic Editor

PLOS One
